# Regulation of tomato fruit elongation by transcription factor BZR1.7 through promotion of *SUN* gene expression

**DOI:** 10.1093/hr/uhac121

**Published:** 2022-05-26

**Authors:** Ting Yu, Guo Ai, Qingmin Xie, Wenqian Wang, Jianwen Song, Jiaying Wang, Jingbao Tao, Xingyu Zhang, Zonglie Hong, Yongen Lu, Jie Ye, Yuyang Zhang, Junhong Zhang, Zhibiao Ye

**Affiliations:** Key Laboratory of Horticultural Plant Biology, Ministry of Education, Huazhong Agricultural University, Wuhan 430070, China; Key Laboratory of Horticultural Plant Biology, Ministry of Education, Huazhong Agricultural University, Wuhan 430070, China; Key Laboratory of Horticultural Plant Biology, Ministry of Education, Huazhong Agricultural University, Wuhan 430070, China; Key Laboratory of Horticultural Plant Biology, Ministry of Education, Huazhong Agricultural University, Wuhan 430070, China; Key Laboratory of Horticultural Plant Biology, Ministry of Education, Huazhong Agricultural University, Wuhan 430070, China; Key Laboratory of Horticultural Plant Biology, Ministry of Education, Huazhong Agricultural University, Wuhan 430070, China; Key Laboratory of Horticultural Plant Biology, Ministry of Education, Huazhong Agricultural University, Wuhan 430070, China; Key Laboratory of Horticultural Plant Biology, Ministry of Education, Huazhong Agricultural University, Wuhan 430070, China; Department of Plant Sciences, University of Idaho, Moscow, ID 83844, USA; Key Laboratory of Horticultural Plant Biology, Ministry of Education, Huazhong Agricultural University, Wuhan 430070, China; Key Laboratory of Horticultural Plant Biology, Ministry of Education, Huazhong Agricultural University, Wuhan 430070, China; Key Laboratory of Horticultural Plant Biology, Ministry of Education, Huazhong Agricultural University, Wuhan 430070, China; Key Laboratory of Horticultural Plant Biology, Ministry of Education, Huazhong Agricultural University, Wuhan 430070, China; Key Laboratory of Horticultural Plant Biology, Ministry of Education, Huazhong Agricultural University, Wuhan 430070, China

## Abstract

Fruit shape is an important biological trait that is also of special commercial value in tomato. The *SUN* gene has been known as a key regulator of tomato fruit elongation for years, but the molecular mechanisms underlying its transcriptional regulation remain little understood. Here, a unique BZR1-like transcription factor, BZR1.7, was identified as a *trans*-acting factor of the *SUN* gene promoter that bound to the conserved E-box of the promoter to promote *SUN* gene expression. Overexpression of *BZR1.7* in tomato led to elevated *SUN* gene expression and formation of elongated fruits. Plants of the *BZR1.7* knockout mutant created by gene editing did not exhibit an observable fruit shape phenotype, suggesting possible functional redundancy of *BZR1*-like genes in tomato. There were seven *BZR1*-like genes in the tomato genome and overexpression of *BZR1.5* and *BZR1.6* led to elongated fruit phenotypes similar to those observed in the *BZR1.7* overexpression lines, further supporting the notion of functional redundancy of *BZR1*-like genes in tomato fruit shape specification. Microscopic analysis revealed that there was a decreased number of cell layers in the fruit pericarp in the *BZR1.7* overexpression lines. These findings offer new insights into the regulatory mechanism by which BZR1.7 promotes *SUN* gene expression and regulates fruit elongation in tomato.

## Introduction

Tomato (*Solanum lycopersicum*) is a significant vegetable crop providing invaluable nutrition for human health. Modern cultivated tomatoes display diverse fruit shapes, which have evolved gradually during the process of domestication and improvement [1, 2]. Today, fruit shape serves as a major criterion for quality evaluation and market classification of fruit-bearing crops. Therefore, it is of great importance to study the genetic and physiological factors regulating tomato fruit shape.

Four main quantitative trait loci (QTLs) regulating tomato elongated fruits have been reported: *OVATE*, *OFP20*, *fs8.1*, and *SUN* [1, 3–6]. The *OVATE* gene, encoding a negative regulatory protein, is a key QTL controlling tomato fruit shape transition from round to pear. The fruit shape variation results from a single-nucleotide polymorphism (SNP) in the second exon of *OVATE*, leading to the premature termination of its translated polypeptide [7]. *OVATE* mutations generally contribute to an elongated fruit phenotype, the degree of elongation depending upon the genetic background [8]. *OFP20* protein belongs to the OVATE family of proteins (OFP). It has been shown that a 31-kb deletion upstream of *OFP20*, 6.5 kb away from the transcription initiation site, causes its reduced expression and the formation of the pear fruitshape phenotype [6]. The *fs8.1* locus has been mapped within a 20-cM region near the centromere and in the middle of the short arm of chromosome 8. Due to the reduced frequency of chromosomal crossovers around the centromere, its exact location has not been identified by map-based cloning [9]. The main function of *fs8.1* is to evenly increase the number of cells in the proximal-to-distal direction of tomato reproductive organs [10]. *SUN* is considered to exert the greatest effect on the elongation of tomato fruit. It encodes a calmodulin binding protein that changes plant hormone abundance and secondary metabolism level, promotes longitudinal division, and inhibits transverse division of the fruit cells [11, 12].

Brassinosteroids (BRs) are a kind of plant-specific steroid hormones that play crucial roles in plant growth and responses to environmental stimuli. They regulate the activities of two vital transcription factors, Brassinazole Resistant 1 (BZR1) and BRI1 EMS Suppressor 1 (BES1), through a signal transduction cascade [13, 14]. BZR1 and BES1 share 88% amino acid identity in their conserved amino-terminal domains; hence they are homolog genes [13, 15]. Besides, both of them modulate the expression of multitudinous downstream genes through binding to the E-box (CANNTG) and the BRRE (CGTGT/CG) *cis*-elements in their promoters [16, 17]. Previous studies revealed that BES1/BZR1 participate in biological processes such as cell elongation [18], cell division [19], ovule and seed development [20, 21], seed maturation [22], anther and pollen development [23], flowering [24], plant architecture [25], and photomorphogenesis [26]. Moreover, *BES1*/*BZR1* genes can be induced by drought [27], cold [28, 29], and salt stress [30], as well as nitrogen starvation [31]. Consequently, they coordinate extensive growth and developmental processes and responses to environmental signals in plants [32, 33]. In *Arabidopsis*, *BES1*/*BZR1* genes have been well characterized and have been implicated in the regulation of root [34], stem [15], and hypocotyl elongation [26, 35–38].

Recently, it has been demonstrated that BES1 and BZR1 are crucial regulators of fruit development and ripening. Heterologous expression of *BZR1-1D* (*AtBES1*) in tomato increases carotenoid accumulation and fruit quality attributes [39], and further isobaric tags for relative and absolute quantitation (iTRAQ) analysis revealed that *BZR1-1D* participates in various ripening-associated processes during tomato fruit ripening [40]. Besides, it has been reported that *DkBZR1* and *2* regulate the genes involved in cell wall degradation and ethylene biosynthesis during persimmon fruit ripening [41]. EjBZR1 binds to the BRRE (CGTGTG) motif in the *EjCYP90A* promoter to suppress its expression, inhibiting fruit cell expansion in loquat [42]. Furthermore, BES1 accelerates fruit softening by transcriptional inhibition of *PMEU1* in tomato [43].

We present here evidence that BZR1.7 activates the transcription of *SUN* and promotes tomato fruit elongation. This is, to our knowledge, the first report of a transcription factor that directly mediates the expression of *SUN*. Meanwhile, as the key regulator of the BR signal transduction pathways, BZR1.7 is, for the first time, suggested to modulate fruit shape determination in plants. Our results also demonstrate that phytohormone BRs probably regulate fruit shape through promoting *SUN* gene expression. Thus, this research work sheds new light on the molecular mechanism underlying fruit development in tomato.

## Results

### BZR1.7 is a *trans*-acting factor of *SUN*

**Figure 1:**
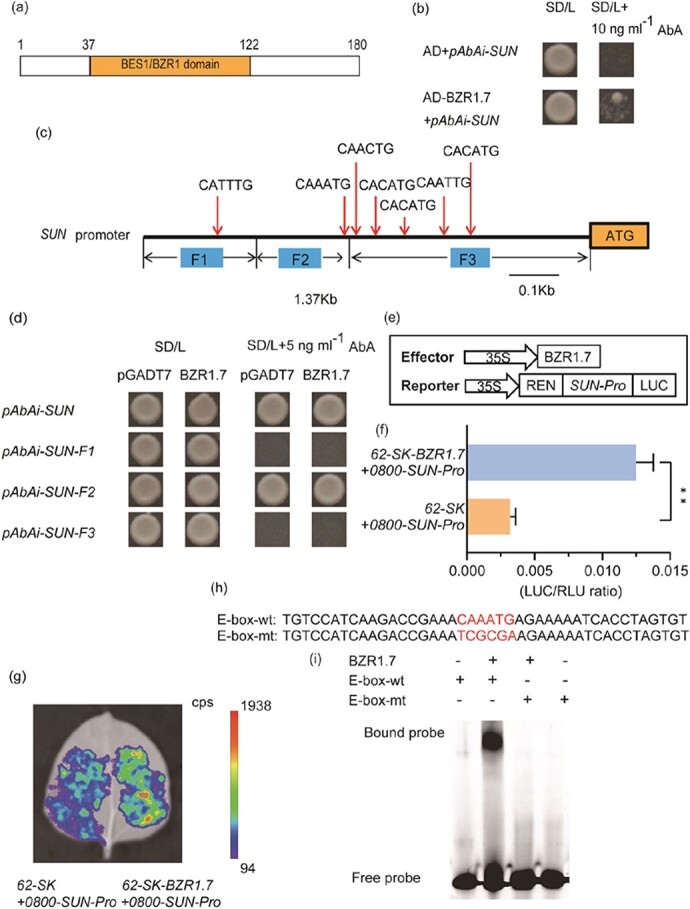
Binding of BZR1.7 to the promoter of *SUN*. **a** BZR1.7 contains 180 amino acid residues, inside which the conserved BES1/BZR1 domain is present between residues 37 and 122. **b** The bait vector pAbAi-SUN and prey vector pAD-BZR1.7 were co-introduced into the yeast strain Y1H Gold. Yeast cells were incubated on (SD/−Leu) without or with 10 ng/mL AbA. Co-transformation of bait vector pAbAi-SUN with the empty vector pGADT7 served as a negative control. **c** The full-length *SUN* promoter (1370 bp) was delineated into three fragments, F1 (from −1370 to −1006), F2 (from −1005 to −761), and F3 (from −760 to 0 bp). Red arrows indicate the locations of the conserved E-box (CANNTG) *cis*-element. **d** The bait vectors pAbAi-SUN (1370 bp), pAbAi-SUN-F1, pAbAi-SUN-F2, and pAbAi-SUN-F3, and prey vector pAD-BZR1.7 were transferred into yeast strain Y1H Gold. Yeast cells were plated on a selective medium (SD/−Leu) without or with 5 ng/mL AbA. Co-transformation of the four bait vectors with pGADT7 served as a negative control. **e** The *SUN* promoter was used to drive the expression of the LUC reporter in pGreen II 0800 LUC and BZR1.7 served as the effector and was expressed from the plasmid pGreen II 62-SK in the dual luciferase assay. **f** A dual-luciferase reporter assay system was used for analysis of BZR1.7 binding to the promoter of *SUN*. The empty vector pGreen II 62-SK was used to replace the effector plasmid in the negative control. Values are expressed as means ± standard deviation (*n* = 6). **g** Transactivation assays tested combinations of BZR1.7 protein and *SUN* promoter construct in *N. benthamiana* leaves. The empty vector pGreen II 62-SK was used to replace the effector plasmid in the negative control. **h** The E-box-wt probe contained a conserved E-box sequence, CAAATG, and this conserved motif was replaced with the TCGCGA sequence in the E-box-mt probe. These probes were labeled with 5′-FAM and used in an EMSA. BZR1.7 was expressed and purified as a recombinant protein (His-6-MBP-BZR1.7). The WT and mutant *cis*-elements are marked in red. **i** Fluorescein-labeled DNA probes were incubated with purified recombinant BZR1.7 and analyzed in EMSA assays.

It has been shown that *SUN* is one of the core genes positively regulating the elongated fruit shape of tomato [11]. For identification of the *trans*-acting factors regulating the expression of *SUN*, a 1370-bp *SUN* promoter fragment was inserted into the pAbAi vector, which was used to transform yeast strain Y1H Gold. Yeast one-hybrid (Y1H) screening was performed via a normalized tomato cDNA library from root, leaf, flower, and fruit tissues at different stages of development. Several proteins, including calmodulin, calcium-binding protein, ethylene-responsive transcription factor, and RIN, were identified as putative *trans*-acting factors for the *SUN* promoter (Supplementary Data Table S1). Based on previous reports and gene annotations, we chose a novel transcription factor gene, designated *BZR1.7*, for further analysis. *BZR1.7*, or *Solyc10g076390*, encodes a member of the BZR1-like proteins and is located on chromosome 10 in tomato. Sequence analysis showed that the gene has a 543-bp open reading frame (ORF) encoding a protein of 180 amino acid residues with a conserved BES1/BZR1 domain located between residues 37 and 122 (Fig. 1a).

**Figure 2:**
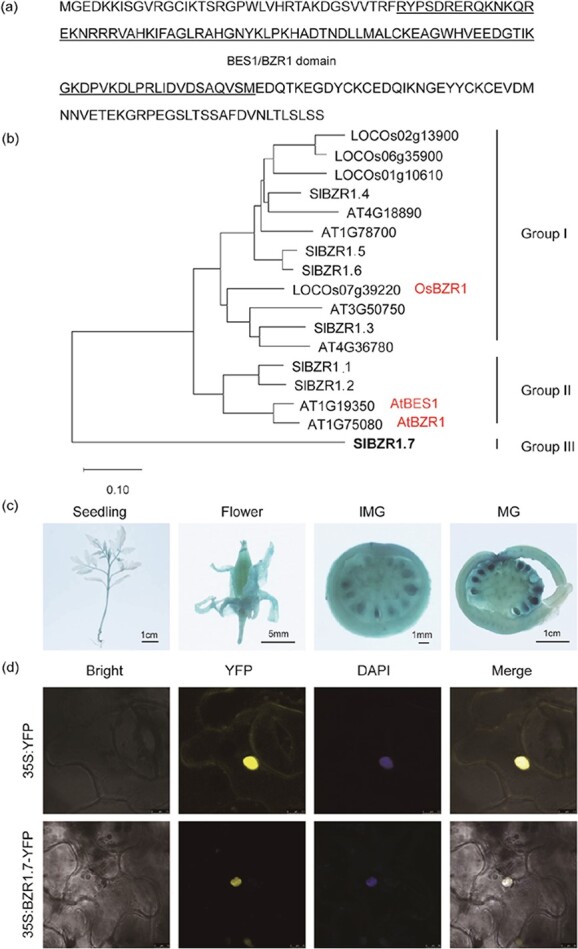
Protein family, spatial gene expression pattern, and subcellular localization of BZR1.7. **a** Amino acid sequence of SlBZR1.7 with the BES1/BZR1 domain underlined. **b** Amino acid sequences of BZR1-like proteins from tomato, *Arabidopsis*, and rice were used for phylogenetic analysis. **c** Spatial expression pattern of the *GUS* reporter gene driven by the *BZR1.7* promoter. IMG, immature green fruit; MG, mature green fruit. **d** Transient expression of 35S:YFP and 35S:*BZR1.7*-YFP in tobacco (*N. benthamiana*) leaves. Images were taken under the bright-field illumination or using fluorescent filters for YFP. DAPI was used to stain the nucleus. Scale bars: top, 7.5 μm; bottom, 10 μm.

To validate the protein–DNA interaction, the Y1H assay was performed. The result showed that BZR1.7 protein was capable of binding to the promoter of *SUN* (Fig. 1b). The PlantCARE website (http://bioinformatics.psb.ugent.be/webtools/plantcare/html/) bioinformatics tool was utilized to analyze the 1370-bp *SUN* promoter sequence [44]. There are seven E-box motifs (CANNTG) in this promoter region (Fig. 1c), but no BRRE (CGTGT/CG) motif is present. To confirm that the E-boxes were important for the transactivation of *SUN*, the promoter was delineated into three fragments, F1 (−1079 to −1370 bp), F2 (−731 to −1078 bp), and F3 (0 to −759 bp) (Fig. 1c), for use in the Y1H assay. The assay result indicated that only F2 showed interaction with BZR1.7, while the other two promoter fragments did not (Fig. 1d), indicating that the *cis*-element recognized by BZR1.7 was located from −731 to −1078 bp in the *SUN* promoter, where the conserved E-box CAAATG motif is present (Fig. 1c). To further confirm the binding of BZR1.7 to the *SUN* promoter *in planta*, we fused the *SUN* gene promoter to a luciferase (LUC) reporter gene and analyzed whether BZR1.7 regulates the transcription of *SUN* via the dual-luciferase reporter assay (Fig. 1e). The result showed that the transactivation activity of BZR1.7 was ~3-fold compared with the empty vector (Fig. 1f). Besides, significantly increased luminescence intensity was observed upon coexpression of BZR1.7 and the *SUN* promoter in tobacco leaves compared with the empty control (Fig. 1g). These data imply that BZR1.7 is able to transactivate the promoter of *SUN*. Thus, we concluded that the CAAATG E-box motif of the F2 fragment is required and sufficient for the BZR1.7-mediated transactivation of *SUN*.

**Figure 3:**
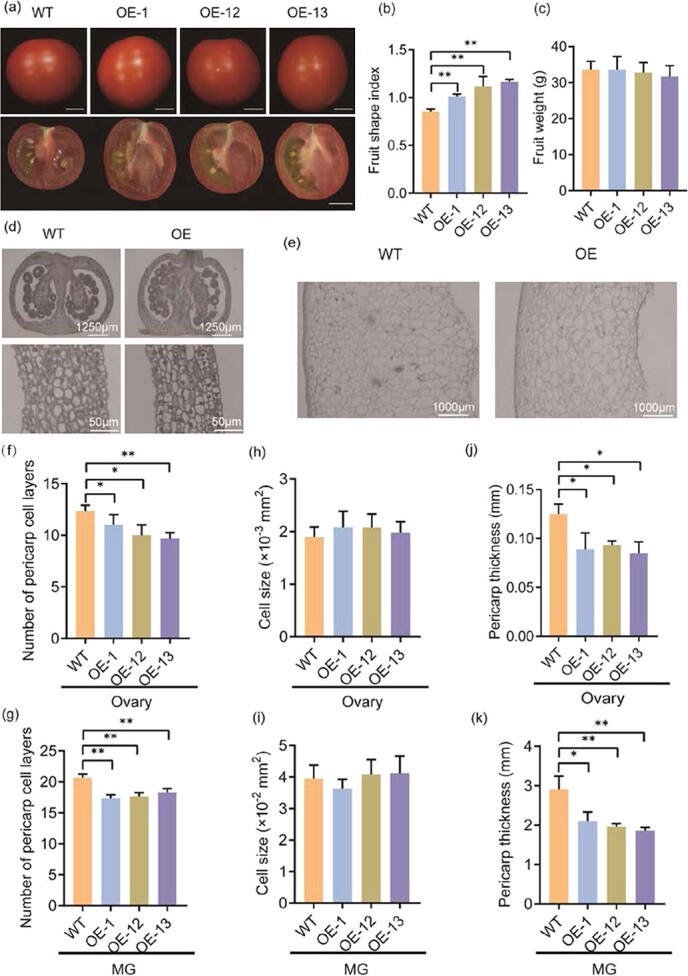
Tomato fruit shape phenotypes of *BZR1.7* OE lines. **a** Longitudinal sections of red ripe fruits from three *BZR1.7* OE lines and their WT control. Scale bar, 1 cm. **b**, **c** Fruit shape index and fruit weight of three *BZR1.7* OE lines and the WT. **d**, **e** Vertical sections of the ovary (**d**) and mature green fruit (**e**) from a *BZR1.7* OE line and its WT control. **f**, **g** Number of pericarp cell layers, **h**, **i** cell size of the parenchyma, and **j**, **k** pericarp thickness of the ovary and mature green fruit (MG), respectively, from the *BZR1.7* OE lines and the WT. ^*^P < .05, ^**^P < .01.

To verify the binding of BZR1.7 to the CAAATG motif of the *SUN* promoter, an electrophoretic mobility shift assay (EMSA) was carried out. A 42-bp double-strand DNA fragment corresponding to the promoter sequence that contained the CAAATG E-box motif was synthesized and labeled with 5′-fluorescein amidite (5′-FAM), a synthetic fluorescein dye (Fig. 1h). The results showed that in the presence of BZR1.7 the 5′-FAM-labeled DNA probe containing the CAAATG *cis*-element shifted to a band of large molecular mass (Fig. 1i, Bound probe). When the DNA sequence in the E-box was changed from CAAATG (E-box-wt) to TCGCGA (E-box-mt), the fluorescence band of large molecular mass was not detected (Fig. 1i), suggesting that the CAAATG sequence of the E-box was required for the binding with BZR1.7 *in vitro*. These data indicate that in plant cells BZR1.7 may regulate the transcription of *SUN* by binding to the CAAATG *cis*-element of the *SUN* promoter.

### Characterization of BZR1.7

A search for conserved domains at the NCBI showed that the amino acid sequence of BZR1.7 contains one BES1/BZR1 domain at the N terminus (Figs 1a and 2a). To explore the evolutionary relationships between the BZR1-like proteins, we constructed a phylogenetic tree for all available BZR1-like sequences from the genomic databases of three representative plant species, including seven sequences from tomato, six from *Arabidopsis*, and four from rice (Fig. 2b). The result illustrated that the majority of the BZR1-like proteins from the three plant species could be divided into three groups. It is impressive to note that tomato BZR1.7 was the only member of an independent branch, and had no close homologs in *Arabidopsis* and rice. This phylogenetic analysis indicates that SlBZR1.7 has probably evolved independently for the function of fruit shape specification in tomato. Analysis of the expression data of *GUS* (β-glucuronidase) activity driven by the *BZR1.7* promoter demonstrated that the GUS staining was observed mainly in the fruit, especially the fruit peel and seeds. Low levels of GUS staining were detected in the young tissues, such as stem tip, stem, leaf axil, leaf vein, and young leaves (Fig. 2c).

To determine the subcellular localization of BZR1.7, tobacco leaves were transfected with a 35S:*BZR1.7*–YFP (YFP, yellow fluorescent protein) fusion construct. The result showed that the yellow fluorescence signal of BZR1.7 was found predominantly in the nuclei and overlapped with the fluorescence signal of DAPI (4′-6-diamidino-2-phenylindole). Nevertheless, the YFP signal of the control vector (35S:YFP) was evenly distributed in the cell (Fig. 2d). This subcellular localization result is in accordance with its function as a transcription regulator.

**Figure 4:**
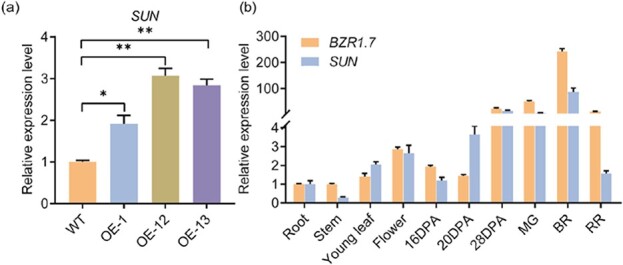
Transcript levels of *SUN* and *BZR1.7*. **a** Transcript levels of *SUN* in the fruits of three *BZR1.7* OE lines and the control. **b** Expression levels of *BZR1.7* and *SUN* in different plant tissues. DPA, day post-anthesis; MG, mature green stage; BR, breaker; RR, red ripe. Transcript levels were normalized to the expression value in roots, which was set at 1. The *actin* gene *Solyc11g005330* was an internal control.

### BZR1.7 promotes tomato fruit elongation

To exploit the biological function of *BZR1.7* in tomato, we first created transgenic plants overexpressing *BZR1.7* (OE lines). The expression levels of *BZR1.7* in the OE lines were quantified via quantitative real time–PCR (qRT–PCR) and were higher than those in the wild type (WT). The averages of the *BZR1.7*-expressing levels were ~2-, 12- and 21-fold higher in OE-1, OE-12, and OE-13, respectively, than those in the WT plants (Supplementary Data Fig. S1). On the other hand, plants of the OE lines displayed marked phenotypic difference in fruit shape. The fruit of *BZR1.7* OE plants was longer than that of the control plants (Fig. 3a). Moreover, fruit widths of *BZR1.7* OE lines were narrower than those of WT while fruit lengths of *BZR1.7* OE lines were longer than those of WT (Supplementary Data Fig. S2). Therefore, the fruit shape index (FSI) was ~20, 30, and 40% higher in OE-1, OE-12, and OE-13, respectively, than that in the control (Fig. 3b). Despite the significant differences in the shape of the fruit, the fruit weight remained unchanged between the OE lines and the WT control (Fig. 3c).

To study the role of *BZR1.7* in fruit shape regulation, we performed microscopic analysis of vertical sections of ovary and mature green fruit of paraffin-fixed tissues (Fig. 3d and e). The *BZR1.7* OE lines showed significant decreases in the number of pericarp cell layers in the ovary compared with the control plants (Fig. 3f). Consistently, the number of pericarp cell layers in the fruit was much lower in the OE lines compared with the control (Fig. 3g). Nevertheless, both the parenchyma cell sizes measured in the ovary and mature green fruit samples were not significantly different between the OE lines and the control (Fig. 3h and i). Marked decreases in pericarp thickness were observed in the ovary and mature green fruit samples in the OE lines compared with the control (Fig. 3j and k). Taken together, these results demonstrate that BZR1.7 promotes tomato fruit elongation.

### Regulation of *SUN* gene expression by BZR1.7

It has previously been indicated that overexpression of *SUN* promotes tomato fruit shape elongation [11]. In order to examine if the binding of BZR1.7 to the *SUN* gene promoter regulates its gene expression, we measured the transcript levels of *SUN* in *BZR1.7* OE lines and recorded an increase of 1- to 2-fold in the three OE lines over the control plants (Fig. 4a). Meanwhile, we investigated the tissue-specific expression of *SUN* and *BZR1.7* via qRT–PCR (Fig. 4b). We discovered that the expression patterns of both *SUN* and *BZR1.7* were very similar and both genes were expressed at high levels in the fruit but low levels in other tissues (Fig. 4b). Therefore, these observations support the notion that *BZR1.7* may directly activate the expression of *SUN* to affect tomato fruit shape.

### Redundant functions of BZR1 family members in tomato fruit shape regulation

**Figure 5:**
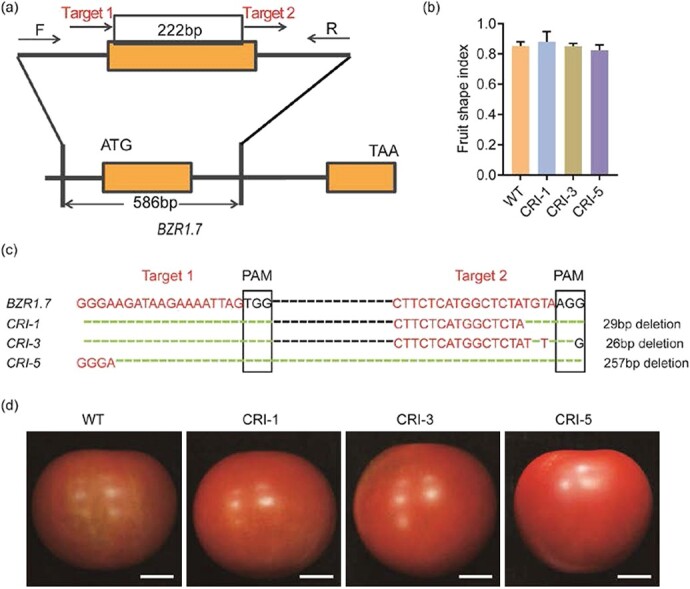
Fruit shape phenotypes of *BZR1.7* knockout lines. **a** Two sgRNA targets (red font) were designed in exon 1 of the *BZR1.7* gene. Black arrows indicate the locations of the primers used to evaluate mutation types in transgenic plants created using the CRISPR/Cas9 technique. **b** Fruit shape index in the BZR1.7 CR lines and the WT control. **c** Genomic DNA sequences of target regions from the WT plant (*BZR1.7*) and three CR lines from the T1 generation. Red letters are the sgRNA target sequences and black boxes represent the protospacer-adjacent motif (PAM) sequences. Black dashed lines represent ellipsis sequences. Green dashed lines represent genomic DNA deletions. CRI, CRISPR/Cas9. **d** Phenotype of red ripe fruit from the three *BZR1.7* CR lines and WT control.

We also generated transgenic tomato lines containing *BZR1.7* gene knockout alleles using CRISPR/Cas9 (*BZR1.7* CR lines) (Fig. 5a). However, the FSI of CR lines did not show notable differences from the WT control (Fig. 5b). Genomic DNA sequencing revealed long deletions and single-base deletions in knockout lines 1, 3, and 5 of the *T*_1_ generation (Fig. 5c). In addition, representative fruit images of the CR lines did not show discernible phenotypes in fruit shape (Fig. 5d). These data suggested that there might be functional redundancy in the *BZR1*-like genes and the effects of the *BZR1.7* gene knockout might be compensated by some of the remaining six members of the *BZR1*-like gene family in tomato (Fig. 2b).

**Figure 6:**
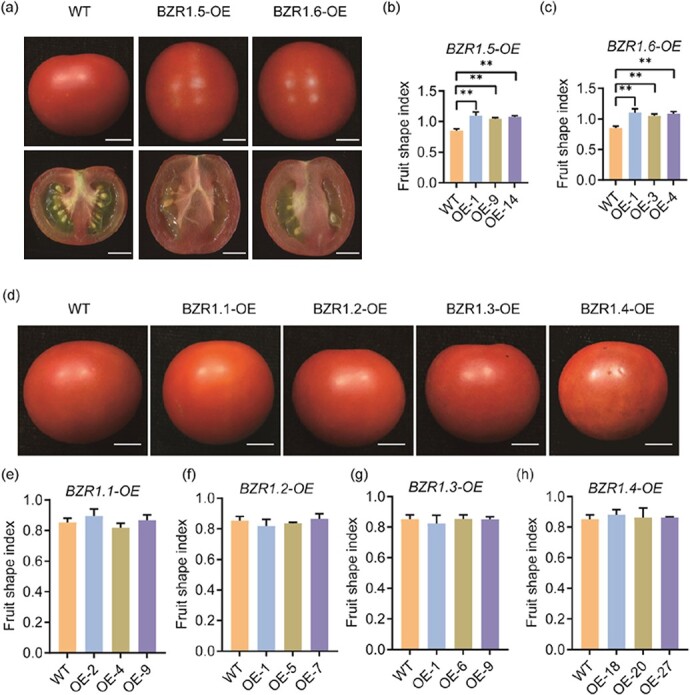
Fruit shape phenotypes of OE lines of tomato *BZR1*-like genes. **a** Fruit shape phenotypes of whole red ripe fruits and their longitudinal sections from representative *BZR1.5* OE lines and *BZR1.6* OE lines. Scale bars, 10 mm. **b**, **c** Fruit shape index of *BZR1.5* OE lines and *BZR1.6* OE lines. **d** Fruit shape phenotypes of whole red ripe fruits from OE lines of the remaining four *BZR1*-like genes. **e**–**h** Fruit shape index of OE lines for *BZR1.1*, *BZR1.2*, *BZR1.3*, and *BZR1.4*.

To explore the functional redundancy of this gene family, we generated transgenic plants overexpressing other *BZR1*-like genes in tomato by stable transformation. The phenotype observations showed that *BZR1.5-*OE lines and *BZR1.6-*OE lines produced fruits that were longer than those of the WT control (Fig. 6a), but similar to those of the *BZR1.7-*OE lines (Fig. 3a). Their FSIs were higher than 1.0, whereas that of the WT fruit was only 0.85 (Fig. 6b and c). It was intriguing to note that the OE lines that overexpressed four other tomato *BZR1*-like genes, *BZR1.1*, *BZR1.2*, *BZR1.3*, and *BZR1.4*, did not exhibit any fruit shape changes (Fig. 6d–h), implying that these genes do not have an effect on regulating fruit shape in tomato. These findings also provided explanations why fruit shape was not affected in the *BZR1.7* knockout lines (Fig. 5b and d). Besides, we also observed that the expression levels of *BZR1.7* in the fruits was reduced remarkably in *BZR1.5-*OE lines and *BZR1.6-*OE lines (Supplementary Data Fig. S3), while the expression levels of *SUN* in the fruits were notably increased (Supplementary Data Fig. S4). The results indicate that there seems to exist a feedback inhibition loop that maintains the total level of the three transcription factors BZR1.5, BZR1.6, and BZR1.7 at a certain level, so that tomato fruit shape would be changed to some extent but would not become too extreme. In summary, there are three BZR1-like proteins, BZR1.5, BZR1.6, and BZR1.7, that perform redundant functions in regulating fruit shape in tomato. In addition, these transcription factors may also regulate each other’s gene expression to keep the total transcript levels of the three genes at a certain level.

**Figure 7:**
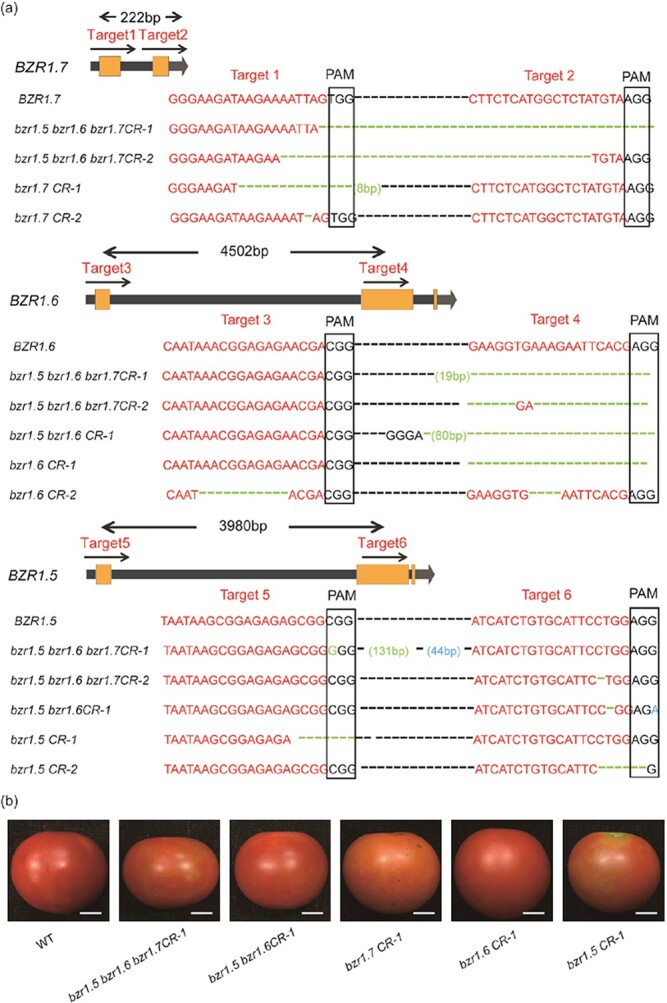
Fruit shape phenotypes of knockout mutants of three *BZR1*-like genes (*BZR1.5*, *BZR1.6*, and *BZR1.7*). **a***BZR1.5* and *BZR1.6* mutations generated by CRISPR/Cas9 as well as *BZR1.7*. Two sgRNA targets (red font) were designed in exon 1 and exon 2 of these genes. The sequences of two *bzr1.7* mutants, two *bzr1.6* mutants, two *bzr1.5* mutants, one *bzr1.5 bzr1.6* mutant, and two *bzr1.5 bzr1.6 bzr1.7* mutants are compared with WT from the *T*_1_ generation. Red letters are the sgRNA target sequences and black boxes represent the protospacer-adjacent motif (PAM) sequences. Black dashed lines represent ellipsis sequences. Green font represents genomic DNA deletions and blue font represents genomic DNA insertions. CR, CRISPR/Cas9. **b** Representative fruit images of all mutants and WT. Scale bars = 1 cm.

In addition, we generated single null mutants, *bzr1.5*, *bzr1.6*, and *bzr1.7*, the null double mutant *bzr1.5 bzr1.6*, and triple *bzr1.5 bzr1.6 bzr1.7* mutants using CRISPR/Cas9 in the AC background (Fig. 7a). Intriguingly, flat fruits were observed in all double and triple mutants. Besides, the *bzr1.5 bzr1.6 bzr1.7* triple mutant had strong fruit shape phenotypes, yet *bzr1.5*, *bzr1.6*, and *bzr1.7* single mutants did not show any apparent difference compared with the WT (Fig. 7b). The detailed FSIs are provided in Supplementary Data Fig. S5a. The expression level of *SUN* was reduced in in all mutants and sharply declined in triple mutants (Supplementary Data Fig. S5b). These results suggested that *BZR1.5*, *BZR1.6*, and *BZR1.7* may have at least partially redundant functions in controlling tomato fruit elongation.

## Discussion

BES1 and BZR1 are two key transcription factors originally identified in the BR signal transduction pathway in *Arabidopsis* and their orthologs and paralogs have since been implicated in multiple facets of plant growth and the development of other plant species [16, 32, 45]. BRAVO is a cell-specific repressor of quiescent center (QC) cell proliferation in the *Arabidopsis* primary root. BES1 represses and interacts with BRAVO, modulating QC cell proliferation at the root stem cell niche and stimulating columella stem cell differentiation [19]. It has been demonstrated that BZR1 induces cell divisions in the QC, but represses cell differentiation at the columella stem, contrary to the action of BES1 [46], suggesting a possibility that BES1 and BZR1 may regulate each other’s gene expression in a reciprocal manner. In addition to their roles in regulating cell divisions, BES1 and BZR1 have also been implicated in the control of cell elongation. The dominant mutation or overexpression of the *ILI1* gene in rice and its homologous gene *PRE1* in *Arabidopsis* can significantly accelerate cell elongation. Overexpression of the *IBH1* gene leads to dwarfism in *Arabidopsis* and erect leaf phenotype in rice. BZR1 can bind to the *ILI1* and *PRE1* gene promoters, induce their transcription, and inhibit the transcription of *IBH1*. This suggests that BZR1 participates in the regulation of cell elongation [18]. In *Arabidopsis*, BZR1 also mediates the antagonism of auxin and BR to regulate the elongation of root cells [34].

Although BES1 and BZR1 have been well characterized in *Arabidopsis* and rice, their functions in fruit development are largely unknown. In this study, *BZR1.7* was demonstrated to play a vital role in promoting tomato fruit elongation. Recently, it has been reported that the interaction of SlBIM1a with BZH1 (BES1) is negatively correlated with cell size in tomato fruit pericarp, suggesting a role of *BES1* in tomato fruit development [47]. Moreover, it has been revealed that *EjBZR1* is negatively related to cell and fruit size in loquat [42]. In this work, our data indicate that *SlBZR1.7* regulates cell division but not cell size of the fruit in tomato (Fig. 3f–i). Besides, its expression level was not correlated with fruit size (Fig. 3c). Microscopic analysis of tissue sections showed that the pericarp thickness of fruit was less in *BZR1.7* OE plants than in the WT control (Fig. 3j and k). Previous reports have shown that *BZR1-1D* participates in the regulation of tomato fruit ripening [39, 40]. In addition, *SlBES1* has also been revealed to play a role in promoting tomato fruit softening, one of the major processes during fruit ripening [43]. Our analysis of the expression pattern revealed that *BZR1.7* was mainly expressed in developing fruits, especially in the pericarp during the late developmental stage (Figs 2c and 4b). These studies suggest that BES1/BZR1-like transcription factors serve as pivotal regulators of fruit development in tomato.

BZR1 homologs exhibit functional redundancy in regulating tomato fruit shape. Although *BZR1.5* and *BZR1.6* are grouped separately from *BZR1.7* on the phylogenetic tree (Fig. 2b), their truncated BZR1 motif conserved amino terminal domains share high identity compared with other BZR1-like proteins. Our data clearly demonstrated that *BZR1.5* and *BZR1.6*, in addition to *BZR1.7*, played noticeable roles in facilitating tomato fruit elongation (Fig. 6a–c). Besides, the fruit shape of the *BZR1.7* knockout lines was similar to that of the WT. These results indicate that tomato BZR1 has functional redundancy. Coincidentally, *BZR1* genes that share high sequence similarities have been shown to exhibit functional redundancy in *Arabidopsis* [17]. In *Arabidopsis*, single mutants of six *BES1*/*BZR1* genes and combinations of double, triple, and quadruple mutants do not show any noticeable growth defects compared with the WT control [48]. Meanwhile, another previous study has also provided consistent results indicating that BZRs redundantly regulate vegetative tissue growth in *Arabidopsis* [49]. These data show that BZR1 proteins are quite conserved. Intriguingly, there are two tomato β-amylase proteins that contain a BZR1-type domain in their N-termini of proteins. In this work, these two β-amylases were not treated as BZR1-like proteins. During the course of this research, we also observed that plants of the *BZR1.7* OE lines developed leaves with smooth leaf edges and reduced leaf angles compared with the serrated leaf edges and flatter leaf angles of the control plants (Supplementary Data Fig. S6a–c). These leaf phenotypes were significant and deserve further investigation in future.

In previous studies, BZR1 has been shown to contain a BIN2 (Brassinosteroid INsensitive 2) phosphorylation domain with over 20 putative BIN2 phosphorylation sites (S/TxxxS/T), which are the targets of BIN2 kinase, a GSK3-like protein kinase that is implicated in the mediation of BR-regulated gene expression [15, 17, 33, 50]. Interestingly, we have not observed any potential role of BIN2 in the phosphorylation of BZR1.7. Sequence alignment showed that BZR1.7 lacks the putative BIN2 phosphorylation sites S/TxxxS/T (Fig. 2a), suggesting that BZR1.7 is probably not phosphorylated by BIN2. *AtBES1* and *AtBZR1* are two well documented genes in *Arabidopsis*, whereas SlBZR1.7 was found to share only 22% amino acid sequence identity with AtBES1 and 21% with AtBZR1 (Supplementary Data Fig. S7). Phylogenetic analysis further showed that SlBZR1.7 does not cluster together with AtBES1 and AtBZR1 (Fig. 2b). The noticeable sequence divergences among them suggest that *BZR1.7* may have a function that is diverse from that of *AtBES1* and *AtBZR1*.

It has been reported that *BZR1* mediates target genes by binding to the E-box (CANNTG) [16]. In this study, our evidence confirmed that BZR1.7 recognized and bound to the CAAATG motif in the *SUN* promoter (Fig. 1d and i). It has previously been reported that *SUN* is involved in regulating tomato fruit shape, floral organ size, and vegetative development of the plant [12]. These phenotypes of *SUN* overexpression were also displayed in plants of the *BZR1.7* OE lines, indicating that *BZR1.7* may exert its function in fruit shape regulation via modulating the expression level of *SUN*. When the *SUN* gene is expressed under the control of the 35S strong promoter in tomato, its transcription levels are markedly high and the transgenic plants produce extreme phenotypes. In this study, the expression level of *SUN* was increased only 1- to 2-fold in *BZR1.7* OE lines and did not lead to extreme phenotypes (Fig. 4a). Consistent with the result obtained with the *sun* mutant, the seed numbers per fruit of *BZR1.7* OE lines were decreased by almost 40% compared with the WT (Supplementary Data Fig. S8a and b). This result suggested that BZR1.7 might control seed development as well as fruit shape.

Taking the results together, we discovered that BZR1.7 promotes tomato fruit elongation by positively regulating *SUN* gene expression. This is, to our knowledge, the first report on the roles of BZR1-like transcription factors in the regulation of fruit shape in tomato and as the upstream regulators of *SUN* gene expression. In conclusion, our results have revealed a novel regulation mechanism of fruit shape involving BZR1.7 and *SUN* and provided new gene targets for alterations and improvement in fruit shape in tomato breeding programs.

## Materials and methods

### Plant materials and growth conditions

Tomato (*Solanum lycopersicum*) cultivar ‘Ailsa Craig’ (AC, LA2838A) was used for sample collections and stable plant transformation. *Nicotiana benthamiana* was used for transient genetic transformation. All plants used in the experiments were cultivated in a greenhouse at the constant temperature of 25°C. The photoperiod was set at the regime of 16 hours light and 8 hours dark.

### Real-time quantitative PCR assay

Total RNA was extracted from various tomato organs, including roots, stems, leaves, flowers, and fruits of different stages using Trizol reagent (Aidlab, Beijing, China). First-strand cDNAs were synthesized from total RNA using a HiScript^®^ II 1st Strand cDNA Synthesis Kit (Vazyme, Nanjing, China). qRT–PCR was carried out in 384-well blocks with the QuantStudio™ 6 Flex Real-Time PCR System to determine the transcript levels of genes in the WT and transgenic plants. Three biological replicates were performed for all assays. The relative expression of specific genes was calculated using the cycle threshold (Ct) 2^-DDCt^ method [51]. Primer sequences are listed in Supplementary Data Table S2. The *Actin* gene (*Solyc11g005330*) was used as a reference gene [52].

### Vector construction and tomato transformation

The full-length coding sequence of *BZR1.7* was amplified from tomato cultivar ‘Ailsa Craig’ using gene-specific primers and cloned into pHELLSGATE8 vector driven by the cauliflower mosaic virus 35S promoter [53]. Loss of function of *BZR1.7* mutations were created via the CRISPR/Cas9 system as described previously [54]. For the CRISPR/Cas9 multiplex editing method we referred to Xie *et al.* [55]. *Agrobacterium tumefaciens*-mediated transformation was performed to conduct constructs for transformation [56]. Transformed shoots that were regenerated from callus were first screened for antibiotic resistance on Murashige and Skoog medium containing kanamycin, then tested by PCR-based assay. Three homozygous *T*_2_*BZR1.7* overexpression lines, OE-1, OE-12, and OE-13, and three homozygous *T*_1_*BZR1.7* knockout lines, CRI-1, CRI-3, and CRI-5, were used for analyses. The primers used are listed in Supplementary Data Table S2.

### Yeast one-hybrid assay

The Y1H assay was performed according to the instructions of the Matchmaker One-Hybrid Library Construction and Screening Kit (Clontech, http://www.clontech.com/). The 1370-bp *SUN* promoter fragment, upstream from its translation initiation codon ATG, was inserted into the pAbAi bait vector using primers listed in Supplementary Data Table S2. After digestion with BbsI, the bait vector was introduced into yeast strain Y1H Gold to create reporter strains. The strain was transformed with empty vector pGADT7 and selected on SD/−Ura−Leu plates with different concentrations of aureobasidin A (AbA) to identify the proper levels of AbA that could restrain the growth of the bait strain comprising empty vector pGADT7. Then a tomato cDNA library was used to transform the strains containing the bait vector. The transformed yeast cells were selected on SD/−Leu−Ura plates with AbA, as mentioned above. The resulting positive clones were collected and their plasmids sequenced. For verification of *BZR1.7* from the positive clones, its coding sequence was cloned into pGADT7 to generate a prey vector. The prey vector was re-introduced into the reporter strain, and cultured on SD/−Leu−Ura plates at 30°C for 72 hours. The positive clones were selected and diluted in double-distilled water to an OD_600_ of 0.1. The suspension was spotted on SD/−Leu−Ura medium with or without AbA. Subsequently, the plates were incubated at 30°C for 3–7 days. The bait strain containing the empty vector pGADT7 was used as a negative control.

### Dual-luciferase assay

The promoter of *SUN* (−1 to −1370 bp) was inserted into the vector pGreen II 0800-LUC to generate a reporter construct. The full-length *BZR1.7* coding sequence was integrated into the pGreen II 62-SK vector to generate an effector construct. Primers used in this assay are listed in Supplementary Data Table S2. These constructs were transformed into *A. tumefaciens* GV3101 together with the pSoup helper plasmid. Tobacco leaves were agroinfiltrated with the plasmids, and harvested 3 days post-infiltration [57]. The activities of LUC and *Renilla* luciferase (REN) were quantified by the Dual-Luciferase Reporter Assay System (Promega). The ratio of LUC to REN represents the transactivation activity. After spraying tobacco leaves with 1 mM d-luciferin (Promega), we captured images of LUC signals with NightSHADE L985 (Berthold) as previously described [58]. The empty vector pGreen II 62-SK was used as a negative control. Six biological replicates were conducted for each combination.

### Electrophoretic mobility shift assay

The coding sequence of *BZR1.7* without the stop codon was amplified and cloned into pET15dMBP to generate fusion construct His-6-MBP-BZR1.7. The plasmid was transformed into *Escherichia coli* BL21 cells as described previously [59]. The BL21 cells were cultured in 400 mL of Luria–Bertani (LB) liquid medium to OD_600_ = 0.6. Isopropyl-β-d-1-thiogalactopyranoside (IPTG) was added to the medium until a final concentration of 0.5 mM to induce protein expression, and the culture was grown with shaking at 16°C for 16 hours. Nickel-nitrilotriacetic acid magnetic agarose was utilized to purify the recombinant protein.

The 42-bp promoter fragment containing CAAATG was synthesized with the 5′-FAM label (Tianyihuiyuan, Beijing, China). The core E-box CAAATG was replaced by TCGCGA in the mutant probe. The labeled single-stranded oligonucleotide probes and unlabeled reverse-complementary oligonucleotides were incubated in a thermal cycler to form 5′-FAM-labeled double-stranded probes. The reaction conditions were as follows: 95°C for 2 minutes, 75°C for 30 seconds, and the annealing temperature decreased by 1°C every cycle, for a total of 50 cycles. Subsequently, labeled double-stranded probes and the fusion protein were incubated in 20 μL of binding reaction buffer for 40 min at 4°C in the dark. The protein–DNA complexes were separated using 6% non-denaturing polyacrylamide gels. Pre-electrophoresis was performed at 4°C at 100 V for 30 minutes using 0.5× Tris-borate-EDTA as the electrophoresis buffer. Then electrophoresis was performed at 4°C at 80 V in the dark for 1 hour. Images were acquired using a FluorChem M (ProteinSimple).

### Sequence comparison and phylogenetic analysis

BES1/BZR1 homologous amino acid sequences from tomato, *Arabidopsis*, and rice were available from NCBI (https://www.ncbi.nlm.nih.gov/). Multiple sequences were aligned with ClustalX 2.1 [60]. A phylogenetic tree was constructed via MEGA X software [61] using the neighbor-joining method. The bootstrap analysis was carried out using 2000 replicates.

### Subcellular localization

The coding sequence without the stop codon of *BZR1.7* was amplified and cloned into the 101YFP vector [62] under the control of the cauliflower mosaic virus (CaMV) 35S promoter to generate a YFP fusion construct (35S:*BZR1.7*-YFP). The vector was introduced into *A. tumefaciens* GV3101 cells, which were used to infiltrate young tobacco leaves. After 2 days of infiltration, the leaves were evaluated and visualized with a confocal laser scanning microscope (SP8, Leica). DAPI at 5 μg/mL was used to stain the nuclei. 35S:YFP (empty 101YFP vector) was used as a positive control.

### GUS staining

Transgenic plants containing the BZR1.7 promoter:*GUS* gene construct were used for GUS staining analysis using different tissues, including seedlings, flowers, and mature green fruits, with a GUS staining buffer [10% methanol, 1 mM 5-bromo-4-chloro-3-indolyl-β-d-glucuronic acid (X-Gluc), 50 mM NaPO_4_, and 0.5% Triton X-100] [63]. After incubation for 12 hours at 37°C in the dark, a graded ethanol series was used to remove the floating color at room temperature.

### Fruit shape analysis

Red ripe stage fruits were cut longitudinally and scanned at 1200 dpi. The highest fruit height and widest width were measured with a Vernier caliper, and their ratio was used as the FSI [3]. All measurements of fruit samples contained three technical replicates and three biological replicates.

### Ovary and pericarp sectioning

Ovaries (~3 days post-anthesis) and pericarps at the mature green stage fruit were used for paraffin sections. Three biological replicates were performed for every transgenic line and WT fruits. Light microscopy was used for observation and photography of paraffin sections. Six or seven images were captured for analysis. The number of cell layers, parenchyma cell size, and thickness of the pericarp of both WT and transgenic plants were measured by ImageJ [64].

### Accession numbers

The gene sequences were obtained from the Sol Genomics Network (http://solgenomics.net/) using the following accession numbers: *BZR1.1*, Solyc04g079980; *BZR1.2*, Solyc12g089040; *BZR1.3*, Solyc02g063010; *BZR1.4*, Solyc07g062260; *BZR1.5*, Solyc02g071990; *BZR1.6*, Solyc03g005990; *BZR1.7*, Solyc10g076390; *SUN*, Solyc10g079240.

### Statistical analyses

Comparisons between two groups were performed via Student’s *t*-test. The significance of differences compared with the WT plants was calculated using GraphPad 8.0, at *P* < .05 and *P* < .01.

## Acknowledgements

This work was supported by the grants from the National Natural Science Foundation of China (31991182, 31872118).

## Author contributions

Z.Y., J.Z., G.A., and T.Y. planned and designed the research; T.Y. performed the experiments; T.Y., G.A., Q.X., W.W., and J.S. analyzed the data; T.Y., Q.X., W.W., J.W., J.T., and X.Z. conducted fieldwork; T.Y. wrote the manuscript; Z.Y., J.Z., Z.H., Y.L., J.Y., and Y.Z. revised the manuscript.

## Data availability

All data supporting the results of this study can be found in this paper or in the supplementary materials.

## Conflict of interest

The authors declare no competing interests.

## Supplementary data

Supplementary data is available at *Horticulture Research* online.

## Supplementary Material

Web_Material_uhac121
