# Supplementary material for: Regulation of tomato fruit elongation by transcription factor BZR1.7 through promotion of SUN gene expression
Source: Hortic Res. 2022 May 26;9:uhac121. doi: 10.1093/hr/uhac121 (PMC9347012; doi:10.1093/hr/uhac121)
Supplement: Web_Material_uhac121 [file web_material_uhac121.zip › Supporting Information clean.docx]

**Supporting information**

Regulation of tomato fruit elongation by transcription factor BZR1.7 through promotion of *SUN* gene expression

Ting Yu *et al*.


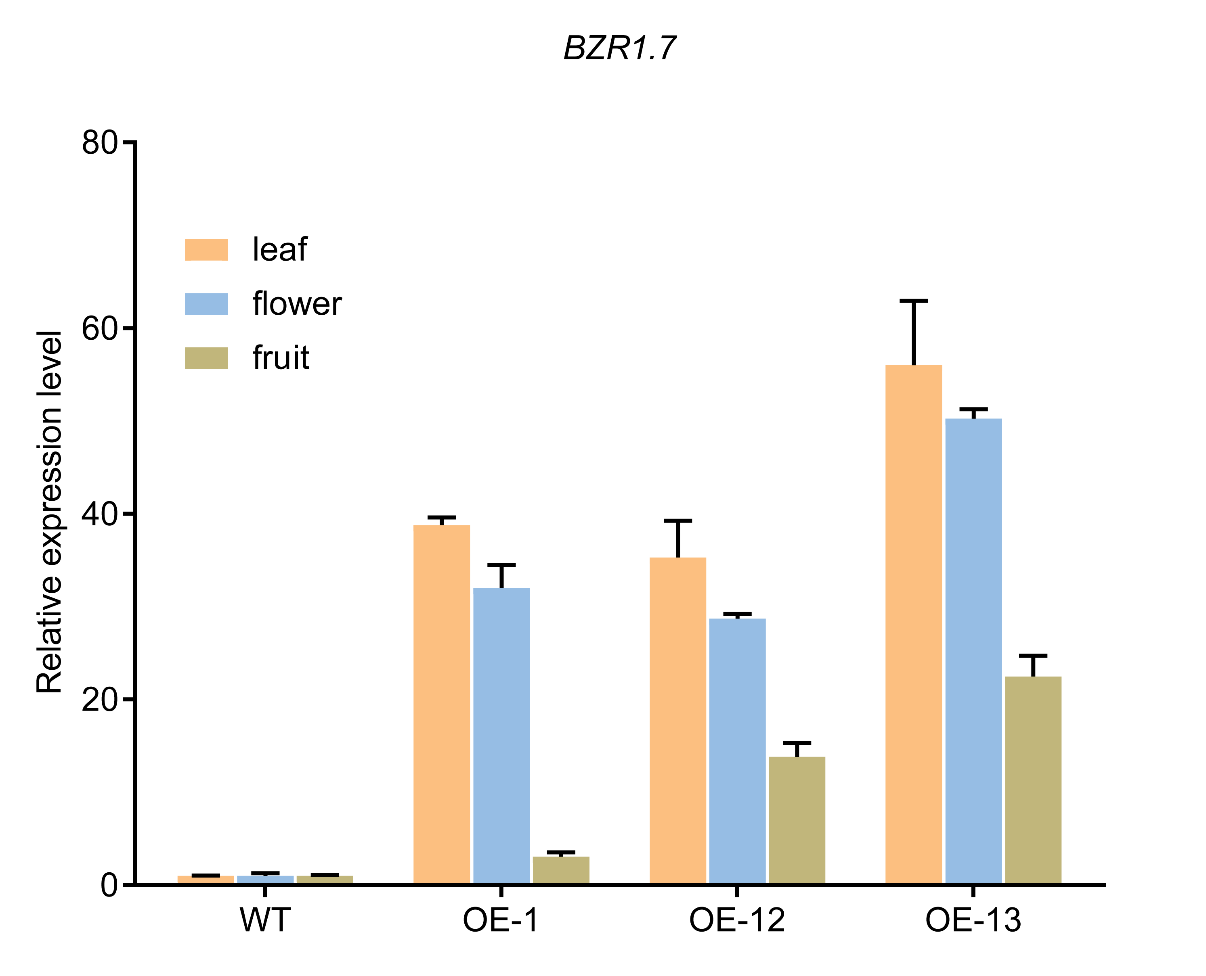


**Figure S1** Transcript levels of *BZR1.7* in leaves, flowers, and fruit from the *BZR1.7* OE lines and the WT control.


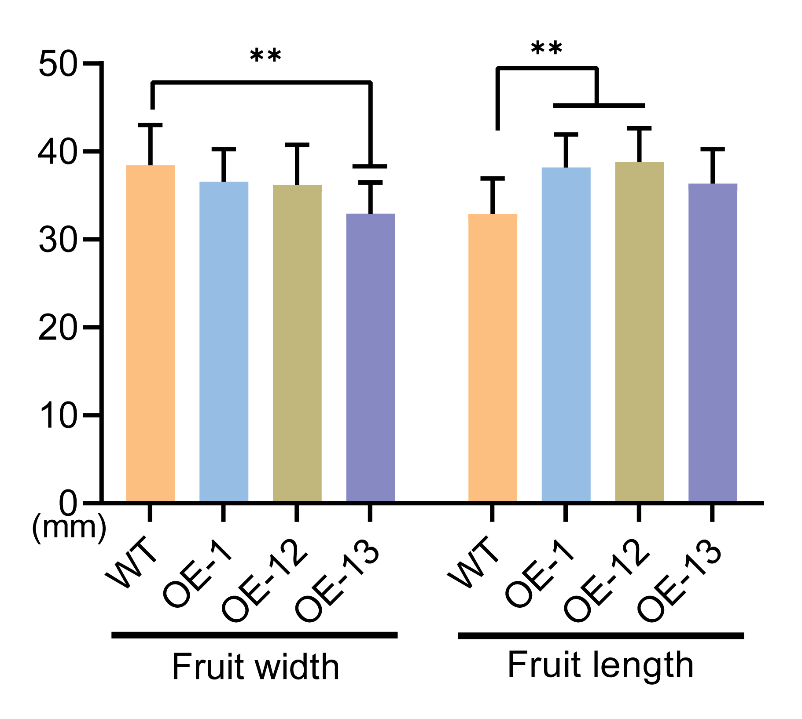


**Figure S2** Fruit width and length of red ripe fruit from the *BZR1.7* OE lines and the WT control (n=10). Statistically significant differences are present by ** at P<0.01.


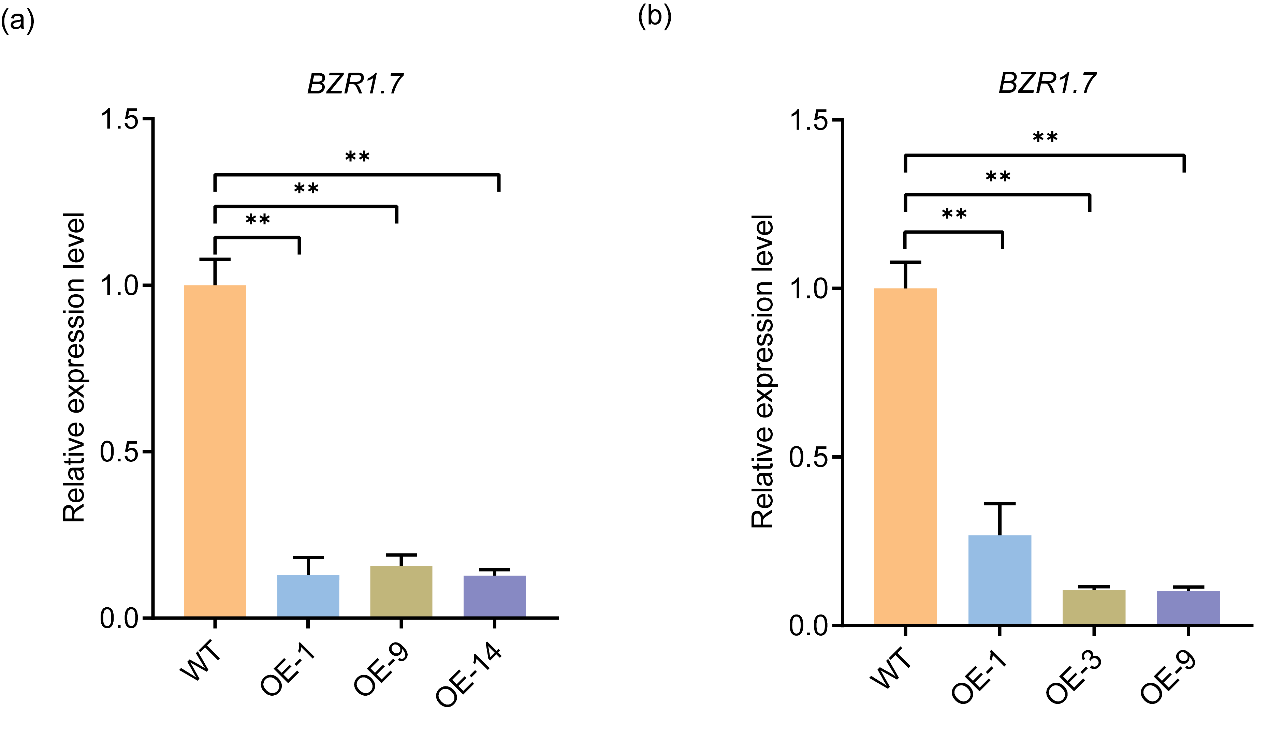


**Figure S3** Transcript levels of *BZR1.7* in the fruit of three *BZR1.5* OE lines (a) and three *BZR1.6* OE lines (b).


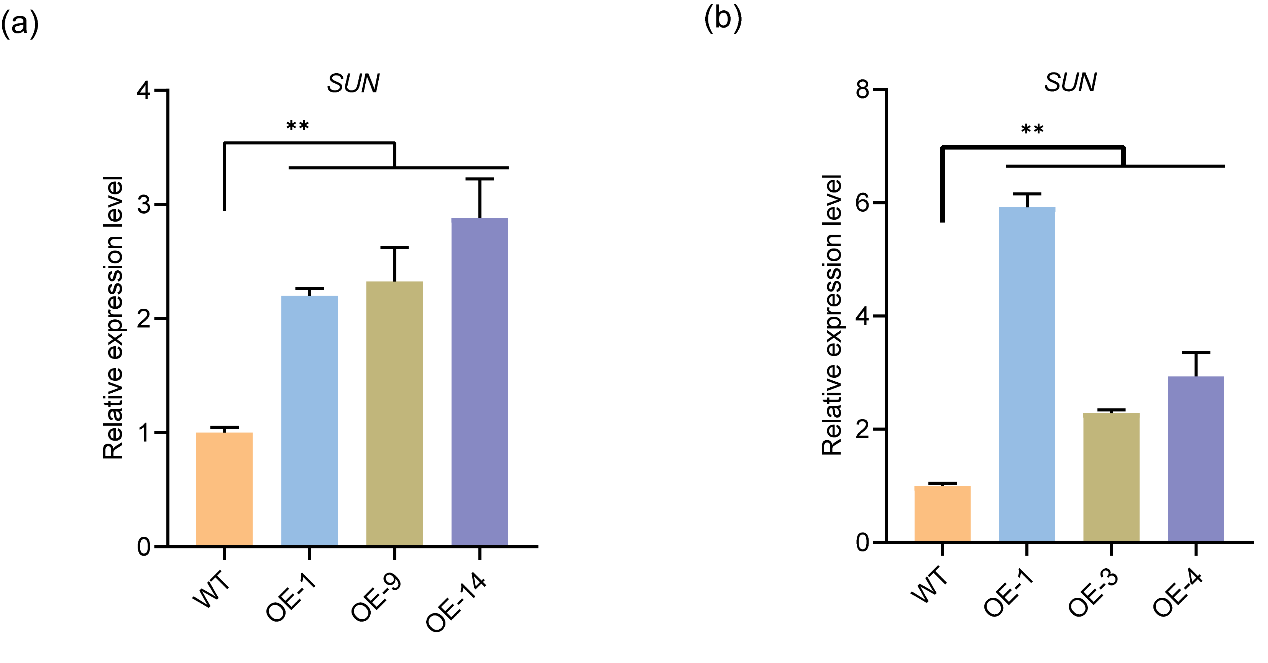


**Figure S4** Transcript levels of *SUN* in the fruit of three *BZR1.5* OE lines (a) and three *BZR1.6* OE lines (b).


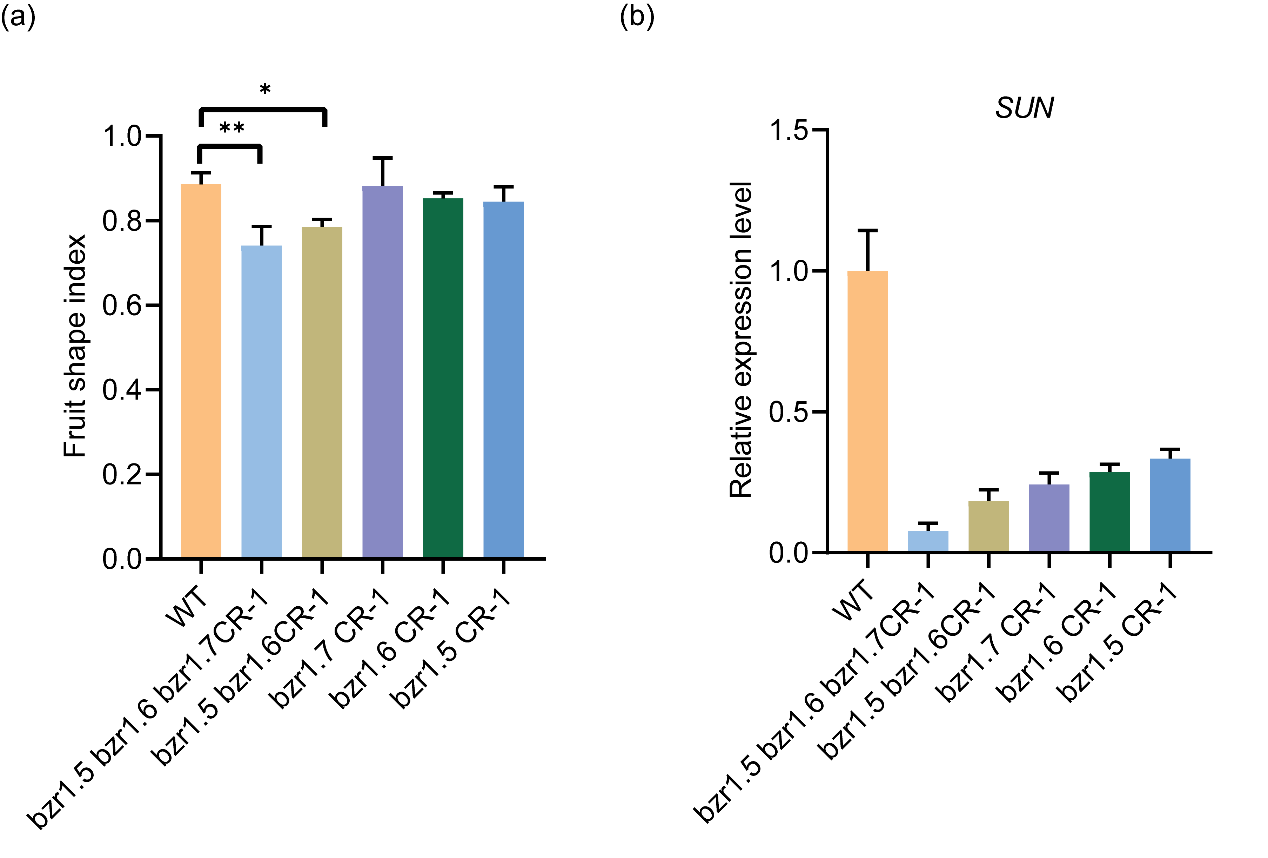


**Figure S5** Fruit shape index (a) and *SUN* expression level (b) of mutants and WT control. Statistically significant differences are established by asterisks ** at P<0.01 and * at P<0.05.

**
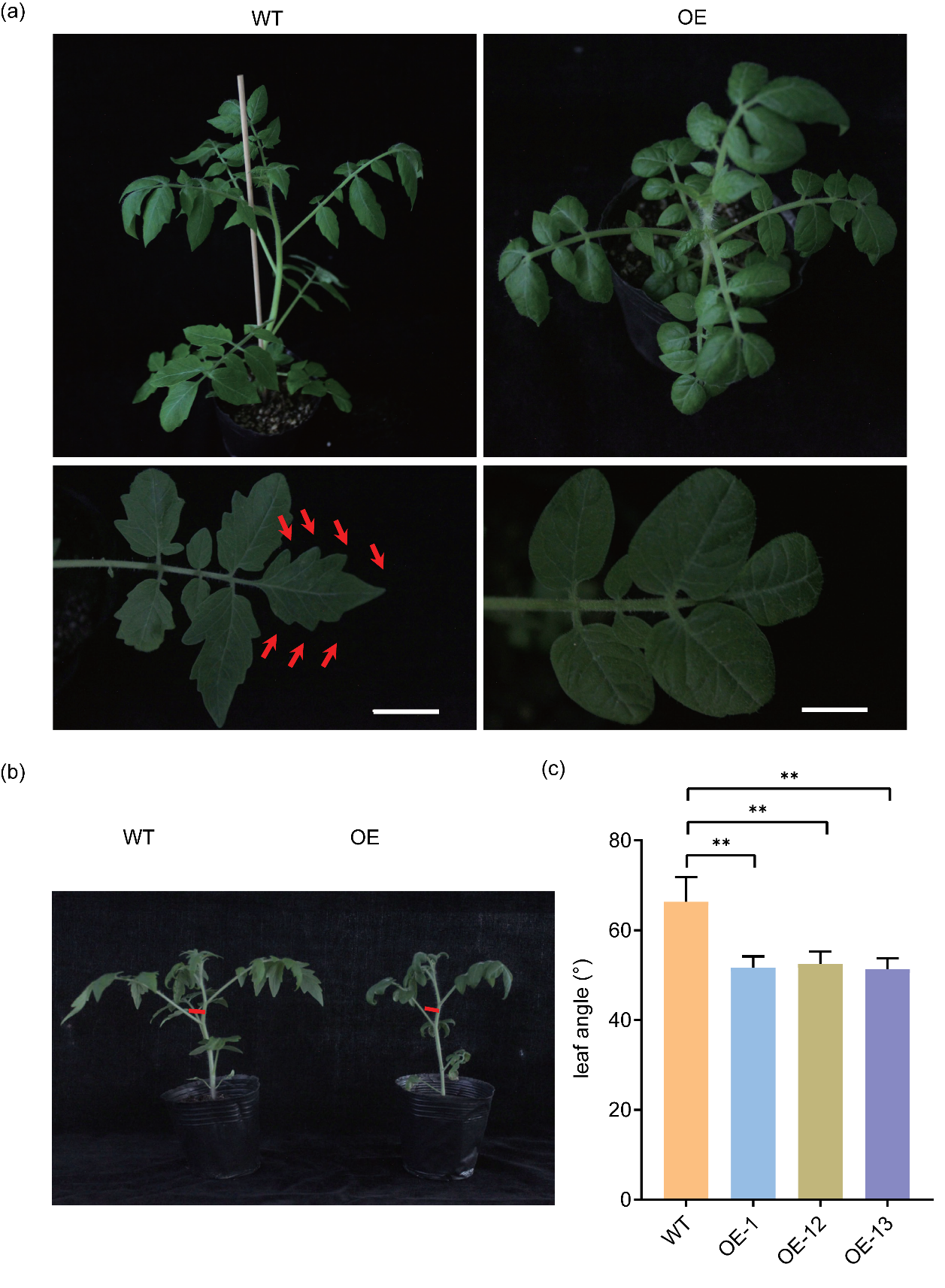
**

**Figure S6** Phenotypes of vegetative tissues of *BZR1.7* OE lines and the WT control. (a) Phenotypes of leaf shape. Red arrows indicate the serrated leaf edge of the control plants. Bars, 10 mm. (b, c) Leaf angles were smaller in the *BZR1.7* OE lines than those in the WT control, as indicated by red lines (b) and degree (^o^) of angle (c). Error bars represent standard deviations for three replicates. Statistically significant differences are established by asterisks ** at P<0.01.


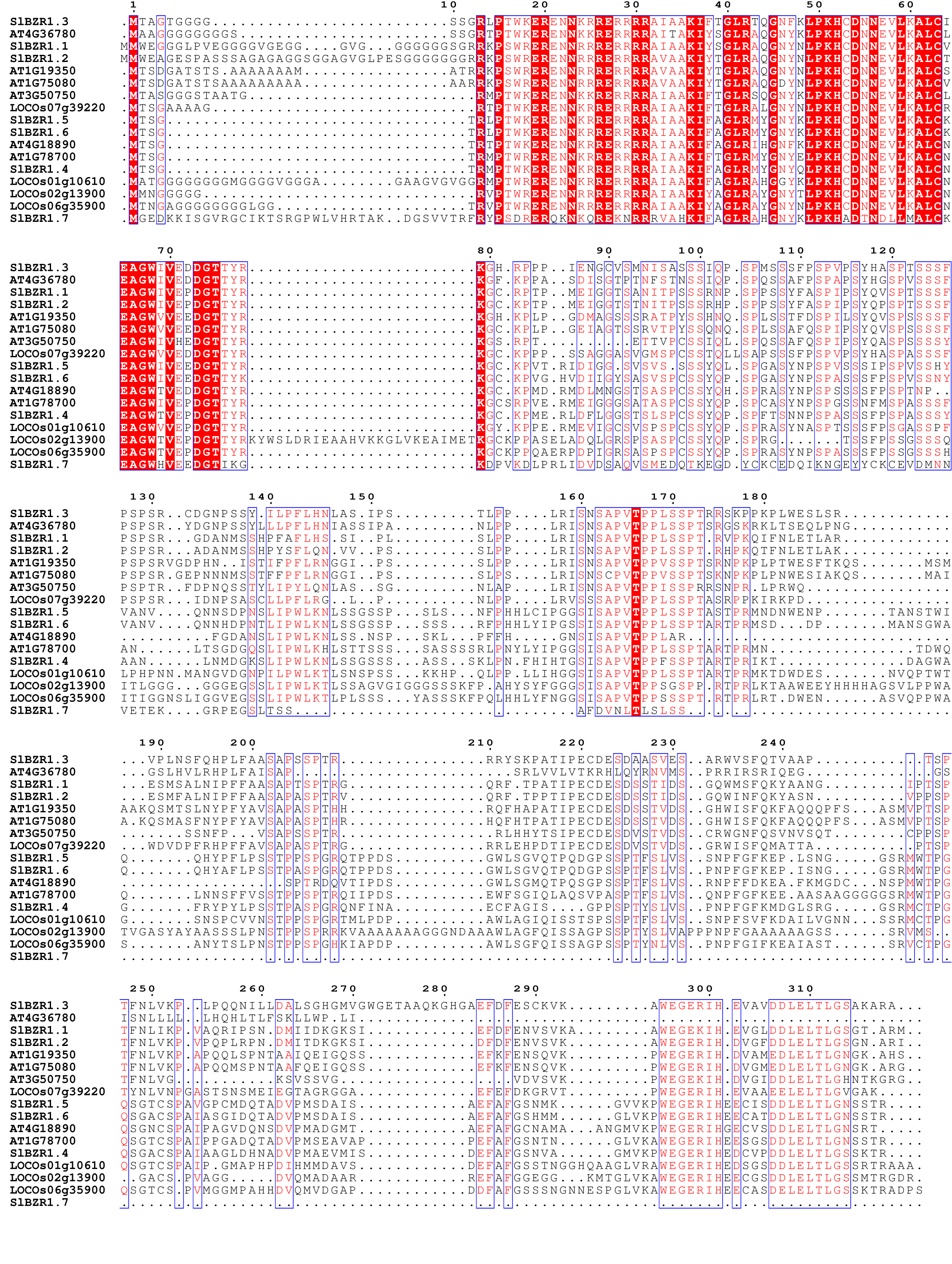


**Figure S7** Amino acid sequence alignment of BZR1-like proteins from tomato, *Arabidopsis* and rice.


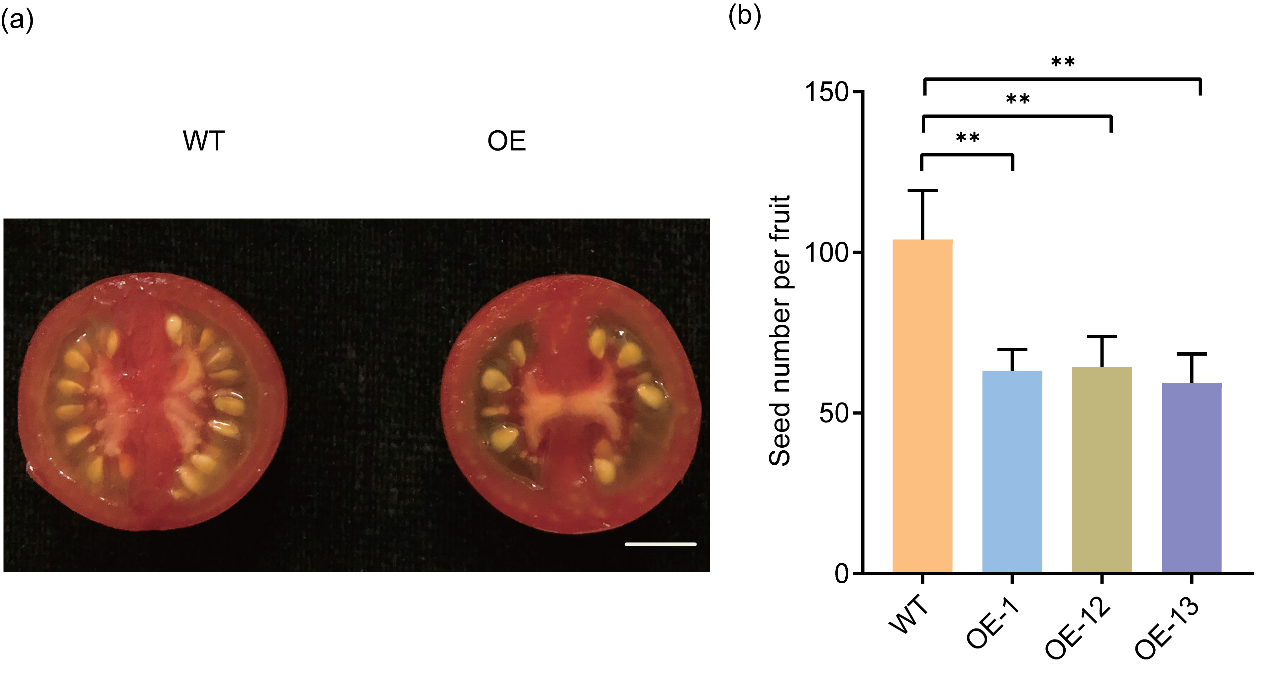


**Figure S8** (a) Cross section of fruit from *BZR1.7* OE lines and WT. (b) Seed number of the *BZR1.7* OE lines as compared with that of the WT control. Statistically significant differences are established by asterisks ** at P<0.01.

| **Table S1 List of genes identified from yeast one-hybrid screen using *SUN* promoter as bait** | |
| --- | --- |
| Gene ID | Annotated Function |
| Solyc10g076390 | BES1/BZR1 homolog protein 1 |
| Solyc10g006130 | Ethylene responsive transcription factor 3a |
| Solyc10g079420 | Calmodulin |
| Solyc10g080770 | BZIP transcription factor |
| Solyc11g012320 | Solanum lycopersicum induced stolen tip protein TUB8-like |
| Solyc10g079120 | Zinc finger CCCH domain-containing protein 30 |
| Solyc02g077880 | Auxin-repressed protein |
| Solyc04g077020 | Tubulin alpha-3 chain |
| Solyc11g012400 | Calcium-binding protein 39 |
| Solyc01g102760 | PHD finger family protein |
| Solyc06g007510 | Ubiquitin-conjugating enzyme E2 8 |
| Solyc03g120500 | Auxin responsive protein |
| Solyc05g056620 | Macrocalyx (mc) Myocyte-specific enhancer factor 2D |
| Solyc05g012020 | Ripening Inhibitor (RIN) MADS-box transcription factor MADS-MC |

| **Table S2 Primers sequences used in the experiments** | | |  | | | | |
| --- | --- | --- | --- | --- | --- | --- | --- |
| Primers | | Sequence(5'-3') | Experiments | | | | |
| OESlBZR1.1-F | | CATTTGGAGAGGACACGCTCGAGTTCTTTTTGGGGAGAAAATGATG Over-expression of BZR1 | | | Over-expression of BZR1 | | |
| OESlBZR1.1-R | | TCTCATTAAAGCAGGACTCTAGATGCTTGAGTGAATCATCAGCTAGC | | |  |  |  |
| OESlBZR1.2-F | | CATTTGGAGAGGACACGCTCGAGATGATGTGGGAAGCTGGAGAATCACCAGCA | | |  |  |  |
| OESlBZR1.2-R | | TCTCATTAAAGCAGGACTCTAGATCATATGCGAGCATTGCCAC | | |  |  |  |
| OESlBZR1.3-F | | CATTTGGAGAGGACACGCTCGAGTTCTCTCTTTTTCTCTCTCCCATTT | | |  |  |  |
| OESlBZR1.3-R | | TCTCATTAAAGCAGGACTCTAGAGTGGTGAACCGCGTGCAA | | |  |  |  |
| OESlBZR1.4-F | | CATTTGGAGAGGACACGCTCGAGCTGCCCATTTGTTGGTTCCA | | |  |  |  |
| OESlBZR1.4-R | | TCTCATTAAAGCAGGACTCTAGATTATCTTGTCTTTGAACTCCCAAGAGTA | | |  |  |  |
| OESlBZR1.5-F | | CATTTGGAGAGGACACGCTCGAGGCGTTGTAATGACTTCCGGC | | |  |  |  |
| OESlBZR1.5-R | | TCTCATTAAAGCAGGACTCTAGACTATCTAGTGCTGGAGTTCCCAAGT | | |  |  |  |
| OESlBZR1.6-F | | CATTTGGAGAGGACACGCTCGAGGCCGATCCAGAACTAAACCG | | |  |  |  |
| OESlBZR1.6-R | | TCTCATTAAAGCAGGACTCTAGACTATCTAGTGCTAGAGTTGCCAAGTGTA | | |  |  |  |
| OESlBZR1.7-F | | CATTTGGAGAGGACACGCTCGAGATGGGGGAAGATAAGAAAATTAGTGG | | |  |  |  |
| OESlBZR1.7-R | | TCTCATTAAAGCAGGACTCTAGATTAAGAAGAGAGTGATAGAGTCAAATTGAC | | |  |  |  |
| pTX-SlBZR1.7-F | | GAATCTAACAGTGTAGTTTGGGGAAGATAAGAAAATTAGGTTTTAGAGCTAGAAATAGC | | Gene editing and detection  (Red font are sgRNA) | | | |
| pTX-SlBZR1.7-R | | GCTATTTCTAGCTCTAAAACTACATAGAGCCATGAGAAGCAAACTACACTGTTAGATTC | |  |  |  |  |
| SlBZR1.7Crispr detection-F | | CAAACTGGAGGAATATATTCTGTCCA | |  |  |  |  |
| SlBZR1.7Crispr  detection -R | ATCATGATGAATCACATTGACACC | | | | |  |  |
| QPCR-SlBZR1.7-F | | GCTCTATGTAAGGAAGCTGGAT | qRT-PCR | | | | |
| QPCR-SlBZR1.7-R  QPCR-SUN-F  QPCR-SUN-R  *Actin* gene-F  *Actin* gene-R | | TTTCTCGGTTTCCACATTGTTC  AGGGCTCTAAAGGGTGTTGTG  CTTCAGTTTGGCAATGAGTCTTC  GTCCTCTTCCAGCCATCCA  ACCACTGAGCACAATGTTACCG |  |  |  |  |  |
| Mul-1-F | | ATATATGGTCTCGTTTGGGGAAGATAAGAAAATTAGGTTTTAGAGCTAGAAATAGC | multiplex editing and detection  (Red font are sgRNA) | | | | |
| Mul-1-R | | ATTATTGGTCTCGGAAGTGCACCAGCCGGGAATCGAA |  |  |  |  |  |
| Mul-2-F | | ATATATGGTCTCGCTTCTCATGGCTCTATGTAGTTTTAGAGCTAGAAATAGC |  |  |  |  |  |
| Mul-2-R | | ATTATTGGTCTCGATTGTGCACCAGCCGGGAATCGAA |  |  |  |  |  |
| Mul-3-F | | ATATATGGTCTCGCAATAAACGGAGAGAACGAGTTTTAGAGCTAGAAATAGC |  |  |  |  |  |
| Mul-3-R | | ATTATTGGTCTCGCTTCTGCACCAGCCGGGAATCGAA |  |  |  |  |  |
| Mul-4-F | | ATATATGGTCTCGGAAGGTGAAAGAATTCACGGTTTTAGAGCTAGAAATAGC |  |  |  |  |  |
| Mul-4-R | | ATTATTGGTCTCGATTATGCACCAGCCGGGAATCGAA |  |  |  |  |  |
| Mul-5-F | | ATATATGGTCTCGTAATAAGCGGAGAGAGCGGGTTTTAGAGCTAGAAATAGC |  |  |  |  |  |
| Mul-5-R | | ATTATTGGTCTCGAAACCCAGGAATGCACAGATGATTGCACCAGCCGGGAATCGAA |  |  |  |  |  |
| BZR1.5Crispr detection-F | | GCAAGCCCGGTCCAGAAGTAAACCGG |  |  |  |  |  |
| BZR1.5Crispr detection-R | | AGAGAGCCATCCTGAATCCGGT |  |  |  |  |  |
| BZR1.6Crispr detection-F | | ATCTTACAAATTTTCAACTTCAAGCC |  |  |  |  |  |
| BZR1.6Crispr detection-R | | AAGCGTTGGTGGACGGTTAG |  |  |  |  |  |
| pMV2-GUS-SlBZR1.7-F | | TGCATCCAACGCGTTGGGAGCTCAACTATTTGCTTTTCGTGTCTCCA | GUS staining | | | | |
| pMV2-GUS-SlBZR1.7-R | | GCCTTCGCCATTCTAGACTCGAGATGCTTCCACTTTTAGTTTATTCAAT |  |  |  |  |  |
| 0800SUN-F | | CACTATAGGGCGAATTGGGTACCAATGTCGCAAATTACCCAACATG | Dual luciferase | | | | |
| 0800SUN-R | | TATGTTTTTGGCGTCTTCCATGGTCAATAGTCCTTCAGCAATCAAACA |  |  |  |  |  |
| 62SKSlBZR1.7-F | | GCCGCTCTAGAACTAGTGGATCCATGGGGGAAGATAAGAAAATTAGTGG |  |  |  |  |  |
| 62SKSlBZR1.7-R | | TTGGTACCGGGCCCCCCCTCGAGTTAAGAAGAGAGTGATAGAGTCAAATTGAC |  |  |  |  |  |
| 101-YFP-SlBZR1.7-F | | ATATGGGATCTACTAGTGAATTCATGGGGGAAGATAAGAAAATTAGTGG | Subcellular localization | | | | |
| 101-YFP-SlBZR1.7-R | | TCGAGCCCGGGGGTACCGTCGACAGAAGAGAGTGATAGAGTCAAATTGAC |  |  |  |  |  |
| ADSlBZR1.7-F | | ACGTACCAGATTACGCTCATATGATGGGGGAAGATAAGAAAATTAGTGG | Yeast one hybrid | | | | |
| ADSlBZR1.7-R | | TACGATTCATCTGCAGCTCGAGCTTAAGAAGAGAGTGATAGAGTCAAATTGAC |  |  |  |  |  |
| pAbai-SUN-F | | AGCTTGAATTCGAGCTCGGTACCAATGTCGCAAATTACCCAACATG |  |  |  |  |  |
| pAbai-SUN-1-R | | ACATACAGAGCACATGCCTCGAGCTTCTTATTAAAGGGCCCTCTGTC |  |  |  |  |  |
| pAbai-SUN-2-F | | AGCTTGAATTCGAGCTCGGTACCATATGATGTTAAAAGAGACTTCCCACA |  |  |  |  |  |
| pAbai-SUN-2-R | | ACATACAGAGCACATGCCTCGAGCGGAGACAAAAAAATACACTAGGTG |  |  |  |  |  |
| pAbai-SUN-3-F | | AGCTTGAATTCGAGCTCGGTACCAAATCACCTAGTGTATTTTTTTGTCTCC |  |  |  |  |  |
| pAbai-SUN-R | | ACATACAGAGCACATGCCTCGAGTCAATAGTCCTTCAGCAATCAAACA |  |  |  |  |  |
| pET15d MBP-SlBZR1.7-F | | TCTGTTCCAGGGGCCGCATATGATGGGGGAAGATAAGAAAATTAGTGG | EMSA | | | | |
| pET15d MBP-SlBZR1.7-R | | TGTTAGCAGCCGGATCCTCGAGTTAAGAAGAGAGTGATAGAGTCAAATTGAC |  |  |  |  |  |
| SlBZR1.7wt-F | | TGTCCATCAAGACCGAAA**CAAATG**AGAAAAATCACCTAGTGT |  |  |  |  |  |
| SlBZR1.7wt-R | | ACACTAGGTGATTTTTCTCATTTGTTTCGGTCTTGATGGACA |  |  |  |  |  |
| SlBZR1.7m-F | | TGTCCATCAAGACCGAAATCGCGAAGAAAAATCACCTAGTGT |  |  |  |  |  |
| SlBZR1.7m-R | | ACACTAGGTGATTTTTCTTCGCGATTTCGGTCTTGATGGACA |  |  |  |  |  |
| The restriction enzyme sites are underlined | | | | | | | |
