# Supplementary figures and images for: Regulation of tomato fruit elongation by transcription factor BZR1.7 through promotion of SUN gene expression
Source: Hortic Res. 2022 May 26;9:uhac121. doi: 10.1093/hr/uhac121 (PMC9347012; doi:10.1093/hr/uhac121)

## Slide 1
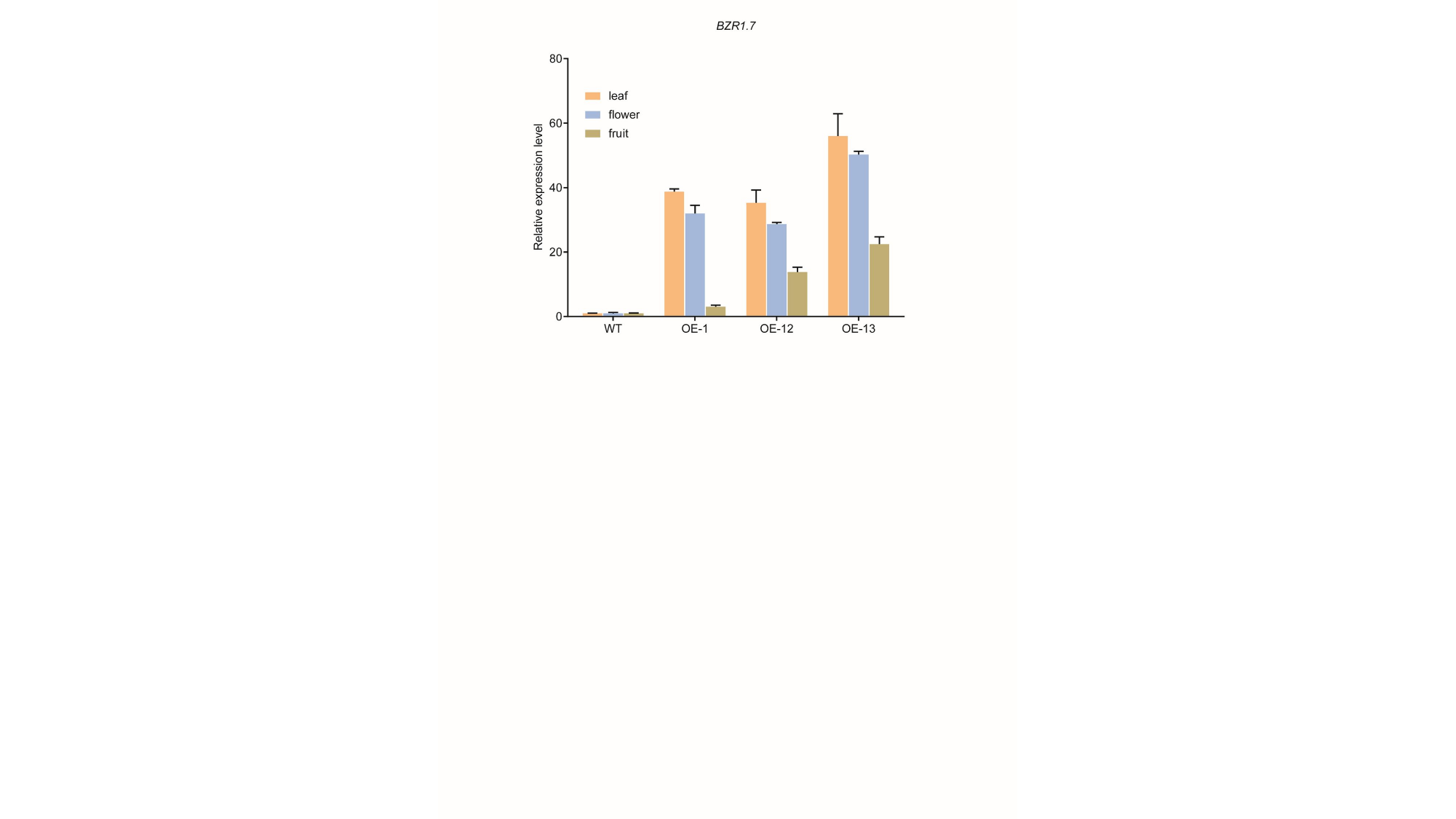

Supplement: Web_Material_uhac121 [file web_material_uhac121.zip › Figure S1.pptx]

## Slide 1
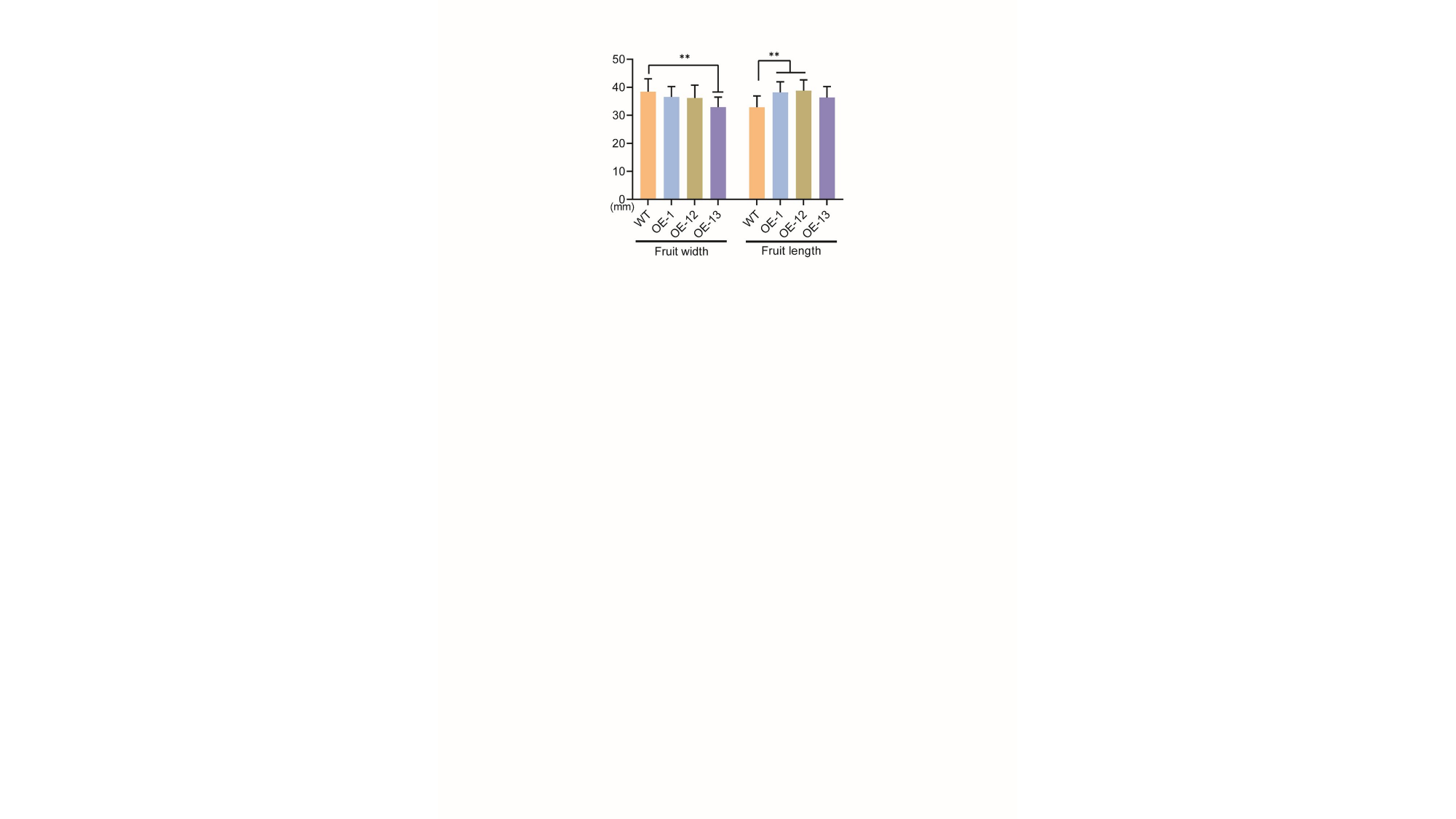

Supplement: Web_Material_uhac121 [file web_material_uhac121.zip › Figure S2.pptx]

## Slide 1
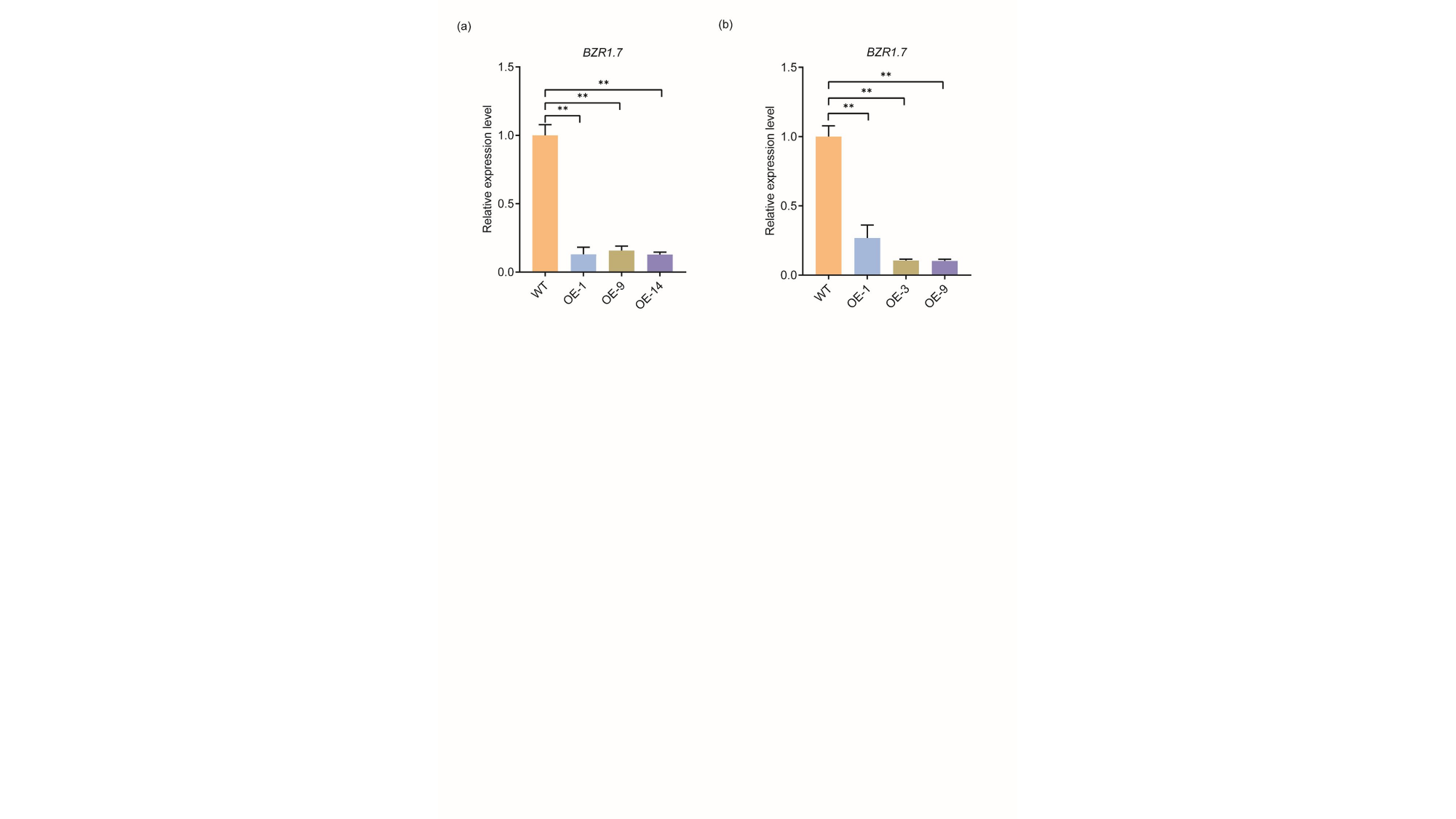

Supplement: Web_Material_uhac121 [file web_material_uhac121.zip › Figure S3.pptx]

## Slide 1
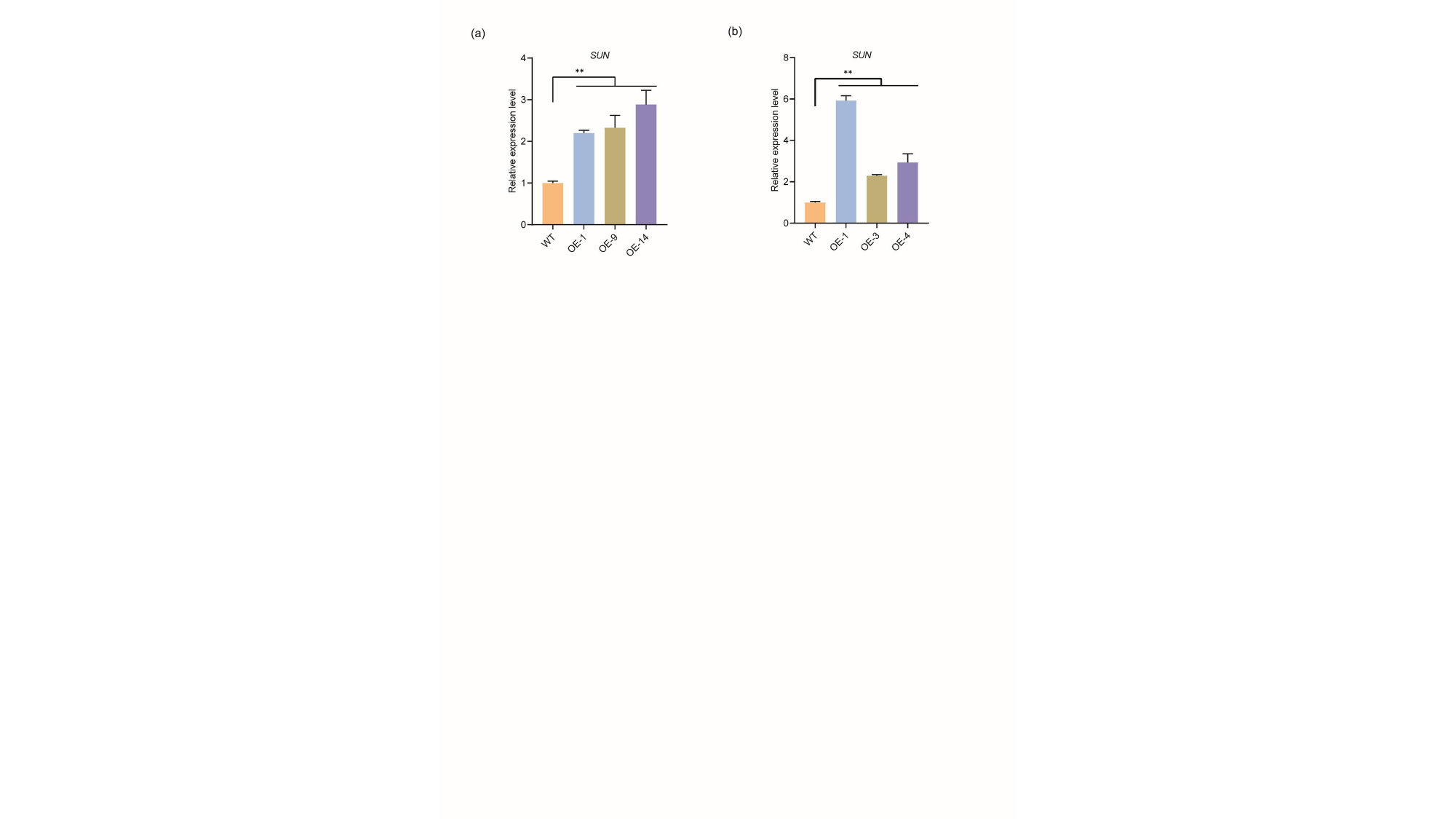

Supplement: Web_Material_uhac121 [file web_material_uhac121.zip › Figure S4.pptx]

## Slide 1
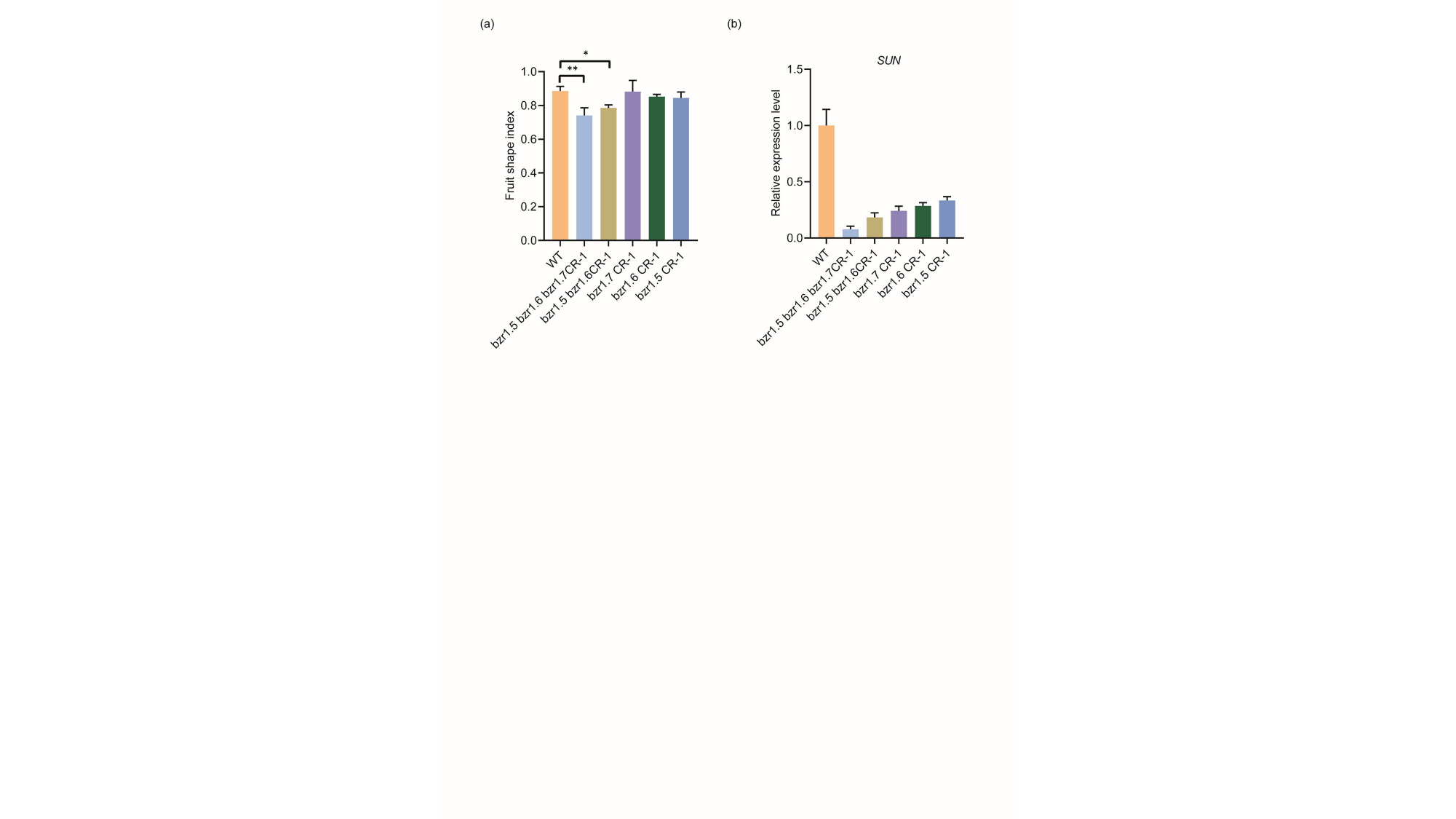

Supplement: Web_Material_uhac121 [file web_material_uhac121.zip › Figure S5.pptx]

## Slide 1
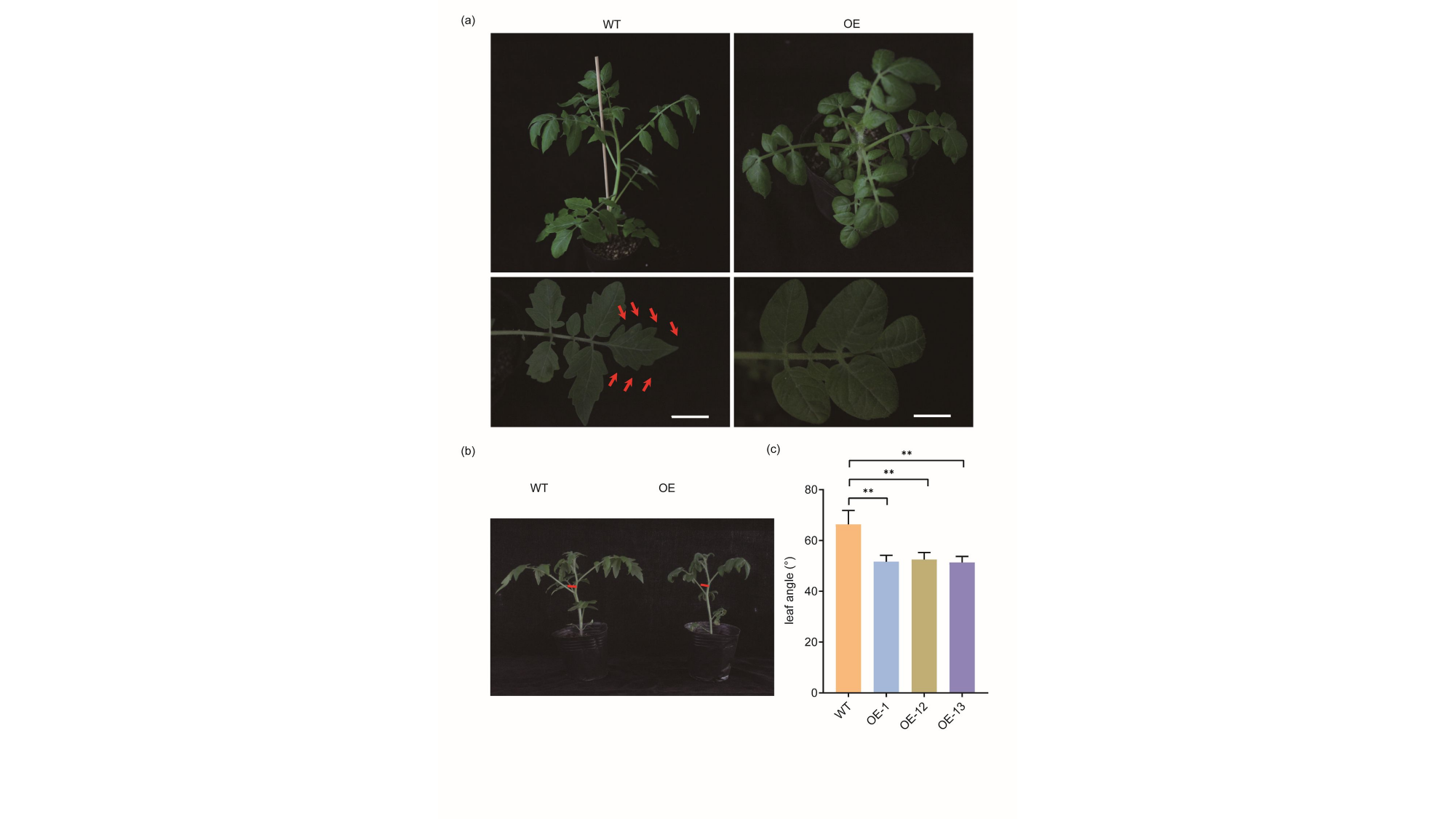

Supplement: Web_Material_uhac121 [file web_material_uhac121.zip › Figure S6.pptx]

## Slide 1
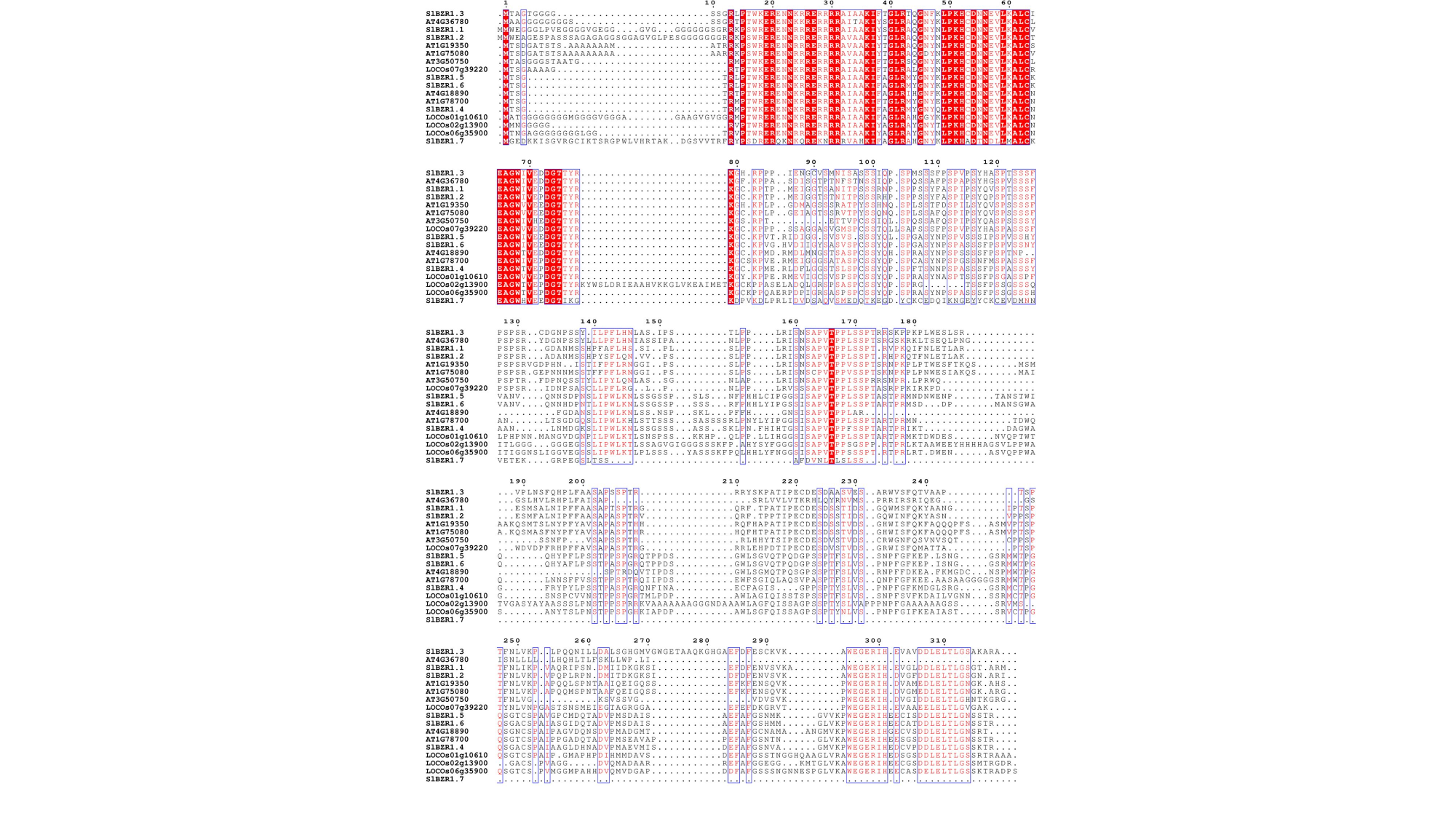

Supplement: Web_Material_uhac121 [file web_material_uhac121.zip › Figure S7.pptx]

## Slide 1
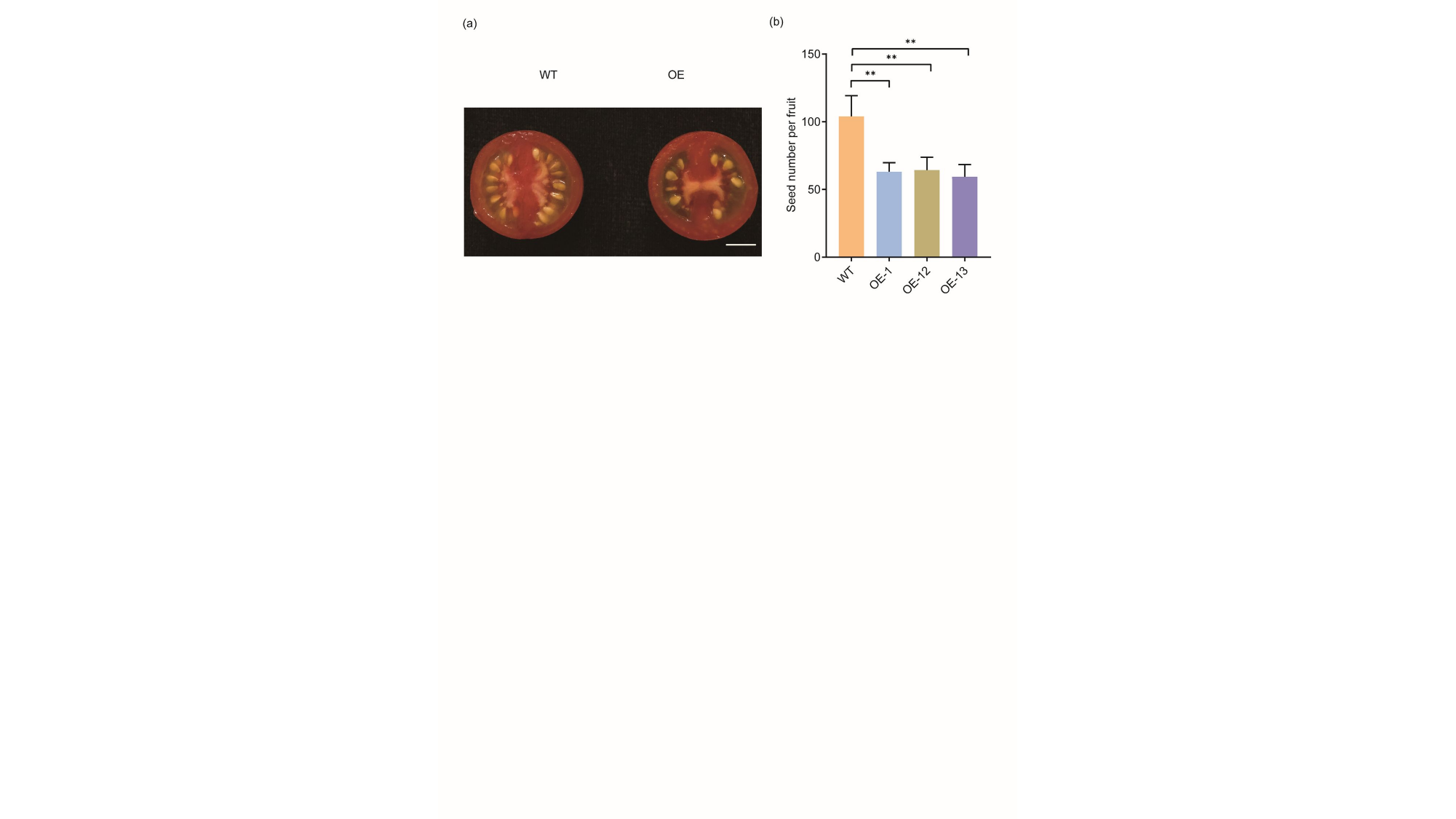

Supplement: Web_Material_uhac121 [file web_material_uhac121.zip › Figure S8.pptx]
